# Liver Gene Expression Profiles Correlate with Virus Infection and Response to Interferon Therapy in Chronic Hepatitis B Patients

**DOI:** 10.1038/srep31349

**Published:** 2016-08-22

**Authors:** Hui-Lin Wu, Tzu-Hung Hsiao, Pei-Jer Chen, Siao-Han Wong, Jia-Horng Kao, Ding-Shinn Chen, Jo-Yang Lu, Tzu-Pin Lu, Yidong Chen, Eric Y. Chuang, Hui-Chu Tu, Chun-Jen Liu

**Affiliations:** 1 Hepatitis Research Center, National Taiwan University Hospital, Taipei, Taiwan; 2 Graduate Institute of Clinical Medicine, National Taiwan University College of Medicine, Taipei, Taiwan; 3 Department of Medical Research, Taichung Veterans General Hospital, Taichung, Taiwan; 4 Greehey Children’s Cancer Research Institute, University of Texas Health Science Center at San Antonio, San Antonio, TX, USA; 5 Department of Internal Medicine, National Taiwan University Hospital, Taipei, Taiwan; 6 Department of Medical Research, National Taiwan University Hospital, Taipei, Taiwan; 7 Graduate Institute of Biomedical Electronics and Bioinformatics, National Taiwan University, Taipei, Taiwan; 8 Bioinformatics and Biostatistics Core, Center of Genomic Medicine, National Taiwan University, Taipei, Taiwan; 9 Institute of Epidemiology and Preventive Medicine, Department of Public Health, National Taiwan University, Taipei, Taiwan; 10 Department of Epidemiology and Biostatistics, University of Texas Health Science Center at San Antonio, San Antonio, TX, USA

## Abstract

The natural course of chronic hepatitis B (CHB) infection and treatment response are determined mainly by the genomic characteristics of the individual. We investigated liver gene expression profiles to reveal the molecular basis associated with chronic hepatitis B and IFN-alpha (IFNα) treatment response in CHB patients. Expression profiles were compared between seven paired liver biopsy samples taken before and 6 months after successful IFNα treatment or between pretreatment biopsy samples of 11 IFNα responders and 11 non-responders. A total of 132 differentially up-regulated and 39 down-regulated genes were identified in the pretreated livers of CHB patients. The up-regulated genes were mainly related to cell proliferation and immune response, with IFNγ and B cell signatures significantly enriched. Lower intrahepatic HBV pregenomic RNA levels and 25 predictive genes were identified in IFNα responders. The predictive gene set in responders significantly overlapped with the up-regulated genes associated with the pretreated livers of CHB patients. The mechanisms responsible for IFNα treatment responses are different between HBV and HCV patients. HBV infection evokes significant immune responses even in chronic infection. The up-regulated genes are predictive of responsiveness to IFNα therapy, as are lower intrahepatic levels of HBV pregenomic RNA and pre-activated host immune responses.

Hepatitis B virus (HBV) infection is an important disease worldwide^1^,^2^. Current therapy for chronic hepatitis B (CHB) relies upon interferon (IFN)-based therapy or nucleos(t)ide analogues^1^. Interferon possesses both antiviral and immunomodulatory effects, and this treatment can effectively suppress and control hepatitis B in 30–40% of HBV-infected patients^3^,^4^. The post-IFN treatment response is more durable than that achieved by nucleos(t)ide analogues, but two thirds of CHB patients respond unsatisfactorily to IFN. The determinants of the IFN treatment response has thus become an important topic to study. In addition, HBV is not directly hepatocytotoxic and the inflammatory changes in liver tissue that characterize CHB are caused by immune responses against the virus. Therefore, it is also very important to understand the immunopathogenesis of hepatocyte inflammation. The natural course of CHB and treatment response of the individual patient will be determined partly by the unique strain of the HBV and mainly by the genetic characteristics of the individual, including polymorphisms and the expressed gene profile^5^,^6^,^7^,^8^,^9^.

In this study, we focused on host liver gene expression profiles in relation to the untreated HBV activity and the response to IFN therapy. Our approach was to analyze the pre-treatment liver gene expression profiles in patients with CHB and compare the IFN responders versus non-responders using Affymetrix Gene Chips. We aimed to identify the gene expression profiles associated with necroinflammatory activity and the profiles potentially predictive of response to IFN therapy for CHB, as these host factors may shed light on the mechanisms of poor response and on the discovery of target molecules or genes for future drug development. Taking advantage of this treatment cohort, we also investigated the paired pre-treatment and post-treatment liver gene expression profiles associated with CHB-related necroinflammatory activities in the IFN responders.

## Patients and Methods

### Clinical characteristics of patients

A total of 38 CHB patients receiving interferon alpha-2b (IFNα) (19 responders and 19 non-responders) in National Taiwan University Hospital were included in this study. These patients received IFNα at a dose of 5 million units thrice weekly for 24 weeks. Patients were followed for an additional period of 6 months post-treatment. The treatment response at the end of the post-treatment 6-month follow-up was defined as HBeAg seroconversion (loss of HBeAg plus presence of anti-HBe), serum HBV DNA level <20,000 IU/mL, and normalization of serum ALT. Those meeting the response criteria were defined as responders, while those not meeting the response criteria were defined as non-responders.

The study protocol conformed to the ethical guidelines of the 1975 Declaration of Helsinki and was approved by the Ethical Committee of the National Taiwan University Hospital (IRB approval number: NTUH-9561703050). Written informed consent was obtained from each patient.

Clinical characteristics of the responders and non-responders were similar in terms of age, gender, serum ALT, and HBV DNA levels (summarized in Table S1). All patients were chronically infected with HBV genotypes B or C and showed evidence of HBV replication and hepatitis within 3 months prior to the study, documented by positive serum e-antigen (HBeAg), serum HBV DNA >20,000 IU/mL, and the presence of alanine aminotransferase (ALT) levels 2- to 10-fold the normal maximum.

### Study design

The liver biopsy specimens of these 38 patients were retrospectively collected for analysis. Seven of 19 IFN responders also had paired post-treatment liver biopsy specimens obtained 6 months after the end of treatment for pairwise analysis (Figure S1A).

The study design is depicted in Figure S1B and the clinical information is listed in Table S2. Two comparisons were performed in this study. The first one compared gene expression profiles between pre- and post- treatment of the responders (BRB vs BRA). The gene expression profiles of the pre- and post-treatment liver biopsy specimens from 7 responders (BRB 1-7 vs BRA 1-7) in the testing dataset, and the profiles of 3 independent responders in the validation dataset (BRB 8-10), were obtained from Affymetrix U133plus2 microarrays.

The second comparison was designed to identify the genes differentially expressed between responders (BR) and non-responders (BN) before treatment. Samples of 11 responders (R7-R17) and 11 non-responders (NR20-NR30) were pooled separately to perform Affymetrix EXON ST 1.0 microarray analysis. The gene expression profiles were submitted to GEO with the accession numbers: GSE66700. The details of sample preparation, experimental protocols, bioinfomatic analysis, and others are described in the Supplemental Materials.

## Results

### Chronic active HBV infection significantly induces intrahepatic gene expression associated with immune responses and cell proliferation

**Figure 1:**
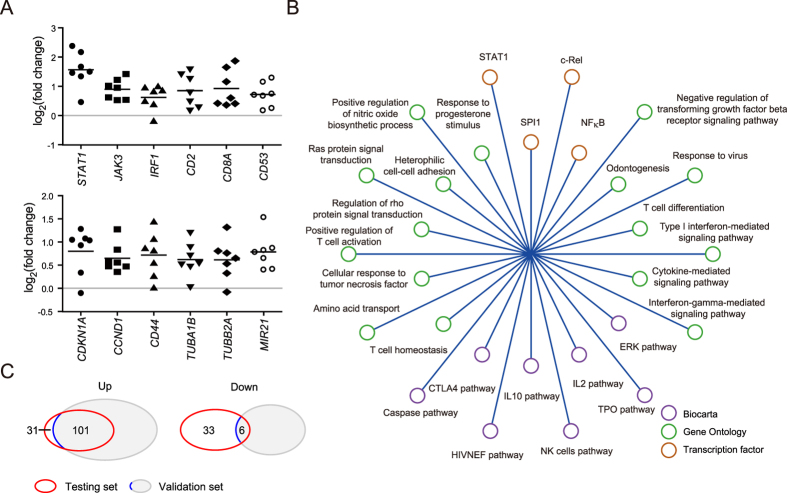
Characterization of the intrahepatic transcription signature associated with necroinflammation of CHB. (**A**) 132 up-regulated and 39 down-regulated genes were differentially expressed between BRB and BRA. Representative genes up-regulated in the BRB samples are depicted. (**B**) GSEA analysis of pathways enriched in the livers of CHB-infected patients. Twenty-eight functions and pathways, which were derived from the gene sets of Biocarta pathway, gene ontology, and transcription factors, were enriched in the BRB samples. (**C**) Venn diagram illustrating the number of significant up- and down-regulated genes in the BRB samples of testing and validation sets.

To identify the genes differentially expressed in the livers of CHB patients, we compared the expression profiles of paired liver biopsies taken before (BRB) and after (BRA) IFNα therapy from 7 responders by microarray. A total of 171 genes (132 up-regulated and 39 down-regulated) were identified to be significantly associated with HBV-related necroinflammation, and these genes are listed in Table S3. Our data showed that immune-related genes were the major genes up-regulated in BRB, such as genes encoding important transcription factors or signal transduction molecules for immune response, CD markers, inflammatory chemokines or chemokine receptors, and human leukocyte antigens (HLA), (Figs 1A and S2). In addition to immune-related genes, some genes involved in liver development and regeneration were also up-regulated in HBV-related inflammatory liver, (Fig. 1A, bottom panel). To move beyond the gene-based approach, we applied Gene set enrichment analysis (GSEA) to identify functions and pathways associated with chronic active HBV infection. A total of 28 functions/pathways were significantly enriched in BRB samples (Fig. 1B). As expected, more than half of them were immune-related. *STAT1*, NF-κB, c-REL, and *SPI1* are 4 important transcription factors regulating immune responses, and their target genes were identified to be enriched. We also found the caspase pathway, growth control signaling, Ras protein signal transduction, and ERK pathways were enriched in HBV-associated necroinflammatory livers.

Although there were a number of genes down-regulated in BRB, no clear pattern was apparent. Moreover, only the up-regulated genes could be validated in an independent set composed of another 3 samples. In the validation set, 76.5% of the 132 up-regulated genes (101/132) also showed differential expression (Fig. 1C). In contrast, only 15.4% (6/39) of the down-regulated genes were validated in the validation set (Fig. 1C). These results indicated that the major difference between HBV inflammatory and non-inflammatory liver tissues was a relative up-regulation of genes in the BRB state.

### IFNγ and B cell signatures are enriched in chronic hepatitis B inflammatory liver

**Figure 2:**
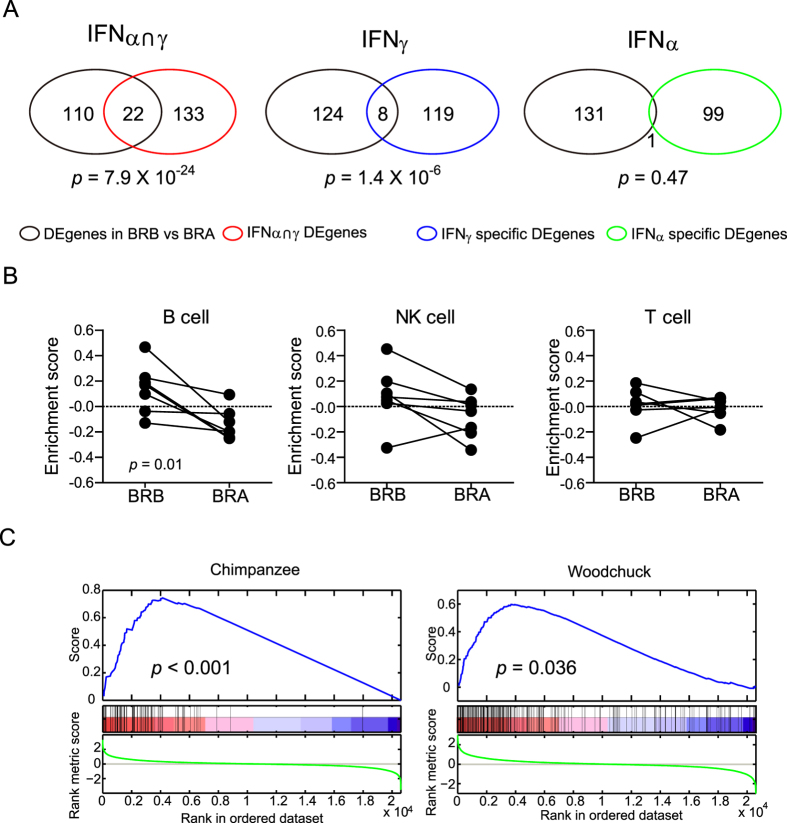
Enrichment analysis of the gene sets associated with IFN, white cell, and animal model. (**A**) Enrichment of IFNγ signatures in CHB-infected patients. Venn diagram illustrates the number of up-regulated genes in BRB samples and IFN signature genes of hepatocytes. (IFNα ∩ γ) indicates genes commonly induced by both IFNα and IFNγ; IFNγ and IFNα indicate genes induced by IFNα or IFNγ, respectively. The *p* values of the enrichment were calculated by Fisher’s exact test. DEgenes: Differential expressed genes **(B**) Enrichment of B cell signatures in CHB-infected patients. The gene signatures of B, NK, and T cells were utilized to estimate the enrichment scores of the respective signatures in paired liver biopsy samples. (**C**) Graphical view of the enrichment of the genes associated with HBV clearance in the chimpanzee model (left panel) and the up-regulated genes in WHV-chronically infected woodchucks (right panel) in BRB samples.

Interferon response is one of the major strategies of the host to combat viral infection. To characterize the interferon responses in CHB, we analyzed the enrichment of interferon-induced genes in the up-regulated differentially expressed genes of the BRB state by Fisher’s exact test. Genes commonly induced by both IFNα and IFNγ (IFNα ∩ γ) were significantly enriched in the BRB state (*p* = 7.9 × 10^−24^). Further analysis revealed that genes preferentially induced by IFNγ (*p* = 1.4 × 10^−6^) but not IFNα were significantly overlapped with the up-regulated genes of the BRB state (Fig. 2A). Results of GSEA were consistent with the Fisher’s exact test (Figure S3). These analyses indicated that the interferon response in the liver of CHB patients is mainly induced by IFNγ but not IFNα. A previous study in the woodchuck model of CHB reached the same conclusion^10^, suggesting that similar IFN responses were induced by HBV and woodchuck hepatitis virus (WHV) in their natural hosts.

To further inspect the immune cell types involved in HBV-associated inflammation, we utilized 3 immune cell subtype gene sets to investigate the biological origins of immune-related genes that exhibit statistical associations with the up-regulated genes of the BRB state. Only the enrichment score of B cells had statistical significance (*p* = 0.01) (Fig. 2B). The GSEA results also confirmed this discovery (Figure S4).

### Differentially expressed genes associated with hepadnavirus infection in animal models were also enriched in chronic hepatitis B patients

The woodchuck and chimpanzee are 2 clinically relevant animal models for HBV infection in humans. We thus examined if the up-regulated genes associated with hepadnavirus infection in these 2 models were enriched in human HBV infection by GSEA. The results showed that same genes up-regulated in chronically WHV-infected woodchucks^10^ were enriched in our BRB sample (Fig. 2C, right panel). Surprisingly, liver genes associated with HBV clearance in the acutely infected chimpanzee model^11^ were enriched in our chronic BRB samples as well (Fig. 2C, left panel).

### Intrahepatic HBV pregenomic RNA level is associated with IFNα treatment response

**Figure 3:**
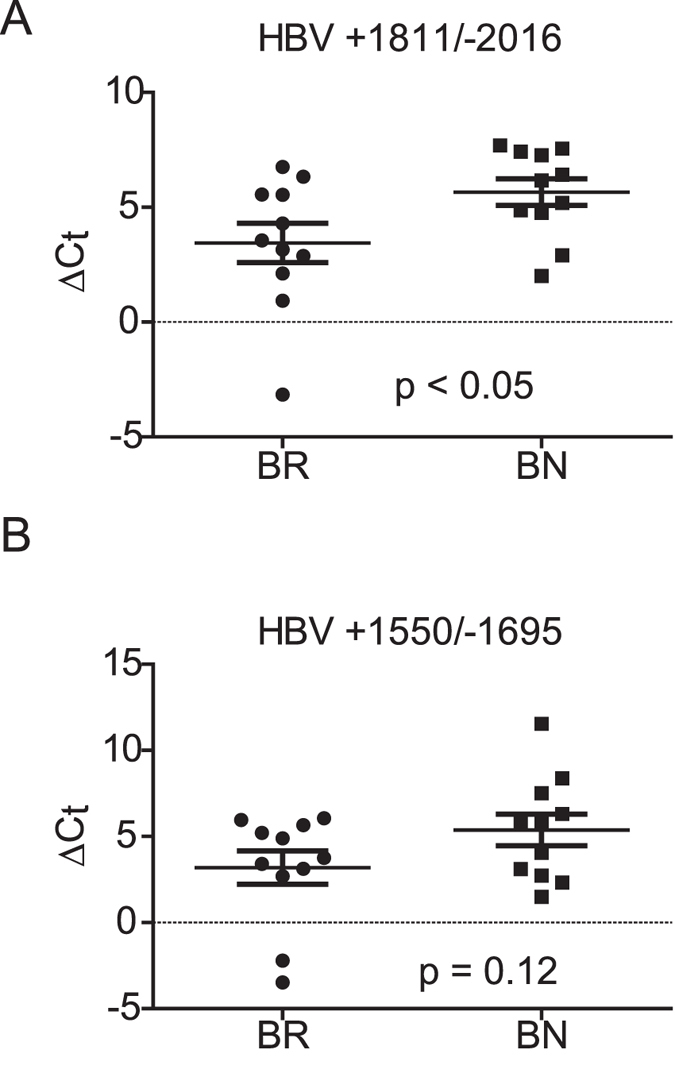
Comparison of intrahepatic levels of HBV pregenomic/precore RNA (**A**) and total transcripts (**B**) between IFNα responders (BR) and non-responders (BN) of CHB. The expression levels of HBV RNA were determined by qRT-PCR with primers HBV +1811/−2016 for pregenomic/precore RNA and HBV +1550/−1695 for total HBV RNA.

IFNα therapy has long been approved to treat patients with CHB; however, only a small proportion of patients respond well to the treatment. The underlying mechanisms which affect the treatment response remain unknown. To discover the factors determining the responsiveness to IFN therapy, we collected liver biopsy samples before treatment from 11 patients responding to IFN treatment (responders, BR) and 11 patients who failed to respond to therapy (non-responders, BN) (Table S1). The serum levels of ALT and HBV DNA did not differ significantly between responders and non-responders (Figure S5). We first examined the intrahepatic levels of HBV pregenomic/precore RNAs and total RNAs by qRT-PCR with primers specific for the core and x regions, respectively (Figure S6). Interestingly, the results showed that the non-responders had higher levels of pregenomic/precore RNA than responders, but the abundance of the total transcripts did not show a significant difference (Fig. 3). This finding was validated in another set of 8 responders and 8 non-responders (Figure S7).

### INF-γ and B cell signatures but not antiviral ISGs are associated with response to IFN therapy

**Figure 4:**
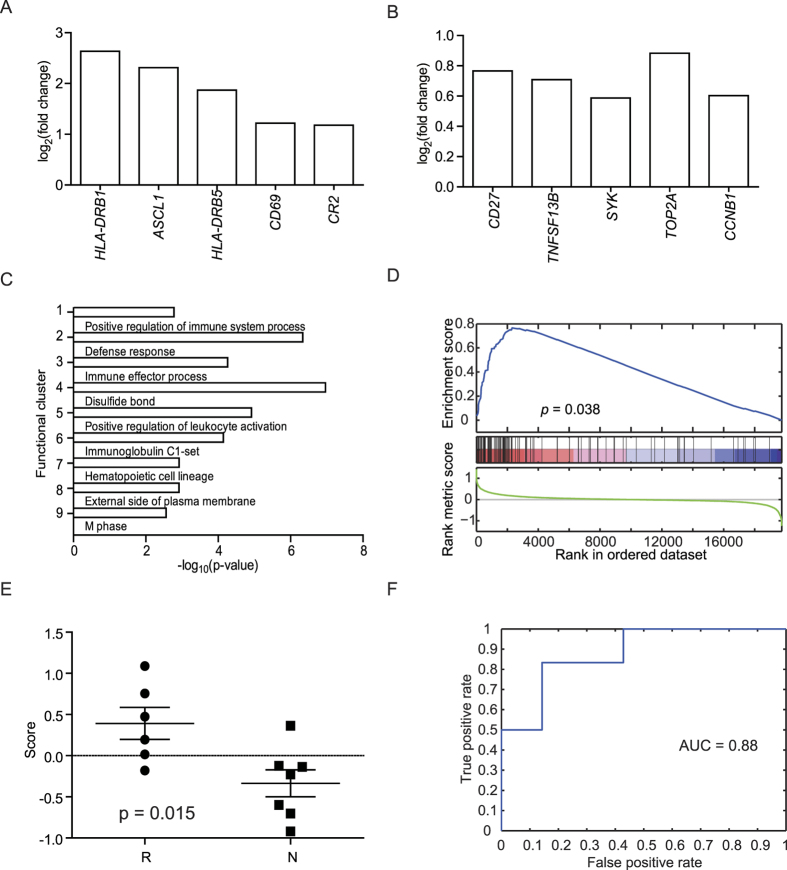
Characterization of the intrahepatic transcriptional signature associated with response to IFNα therapy. A total of 118 up-regulated genes were identified in the comparison of BR and BN. The top 5 (**A**) and other selected significant (**B**) up-regulated genes are depicted. (**C**) Functional annotation analysis of the 118 up-regulated genes in the BR group. (**D**) Graphical view of the enrichment of the 118 up-regulated genes in an independent validation set, GSE27555, by GSEA (*p* = 0.038). (**E**) The enrichment scores of the predictive gene signature, which was constructed by the top 25 up-regulated genes, of responders (R) were significantly higher than those of the non-responders (N) in GSE27555 (*p* = 0.015). (**F**) Receiver operator characteristic (ROC) analysis of the 25 gene signature in GSE27555.

We next analyzed the intrahepatic expression profiles of the 2 groups globally with exon arrays by use of pooled mRNA samples from the 11 responders (BR) and 11 non-responders (BN). A total of 151 differentially expressed genes were identified between BR and BN (118 up-regulated and 33 down-regulated genes in BR) (Table S5). Most of the up-regulated genes in BR are highly associated with immune responses (Figs 4A,B and S8) and Fig. 4A showed the top 5 up-regulated genes. Interestingly, IFN-γ but not IFN-α induced genes, such as *CXCL9*, *CXCL10, GBPs* etc., as well as B cell-related signatures, e.g. *CD27*, *TNFSF13B*, *SYK* and *CR2* etc., are associated with treatment response to IFN-α. In contrast, no significant association of anti-viral interferon stimulated genes (ISGs) with treatment response could be identified in this study. Other immune-related genes, including class II MHC molecules (*HLA-DRB1* and *HLA-DRB5*), *IRF1*, *CR1*, CD antigens, chemokines and genes related to cell proliferation (*TOP2A* and *CCNB1*) were up-regulated as well (Figure S8). The up-regulated genes in responders versus non-responders were further subjected to functional analysis to reveal the mechanisms underlying IFN treatment response. A total of 9 functional clusters were identified (Fig. 4C). Not surprisingly, six of them were correlated with immune functions (the components of the clusters are listed in Table S6. Among them, clusters 3 and 5 contained genes related to B cell response, including “B lymphocyte cell surface molecules” (BIOCARTA) and “Positive regulation of B cell activation” (GO:005087), respectively (Table S6). In addition, the M-phase and cell cycle cluster (cluster 9 in Fig. 4C) was also enriched by the up-regulated genes.

### Up-regulated genes in responders could be the predictors of IFNα treatment response

We validated our data by GSEA with two publicly available independent datasets. The first one, GSE27555, contains the expression profiles of 6 HBV IFN responders and 7 non-responders^12^. The other one, GSE54747, contains profiles of 9 responders and 6 non-responders subjected to IFN and adefovir combined treatment^13^. We found that only the up-regulated genes identified in BR vs BN showed enrichment in these two validation datasets (Figs 4D and S9) and the responders showed higher enrichment scores than non-responders (*p* < 0.05) (Figure S10A). The area under the curve (AUC) in the receiver operating characteristic (ROC) analysis was 0.88 and 0.82 for GSE27555 and GSE54747, respectively (Figure S10B). These data indicate that the up-regulated genes in the BR group could be good predictors for IFNα treatment response.

To make the findings into medical practice, we used the top 25 genes as a predictive gene signature to predict treatment response. As shown in Fig. 4D, the enrichment scores of the signature in BR is higher than BN in the datasets (p < 0.05) (Figs 4E and S11A). The AUC value is 0.88 and 0.80 for GSE27555 and GSE54747 (Figs 4F and S11B). These results indicated that the gene signature could accurately predicted the treatment response of IFNα.

### The genes involved in HBV-related hepatic necroinflammation also play roles in the response to IFNα treatment

**Figure 5:**
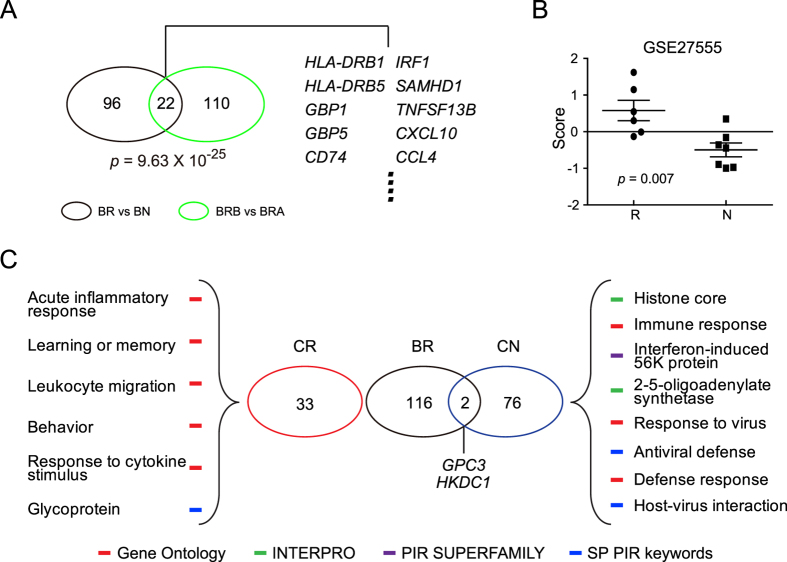
Comparison of the differentially expressed genes associated with IFN treatment response in HBV patients (BR vs BN) to those associated with chronic HBV infection (BRB vs BRA) and to those associated with treatment response in HCV patients (CR vs CN). (**A**) Venn diagram demonstrating the significant overlap between the differentially up-regulated genes in BR vs BN and BRB vs BRA. (**B**) The responders (R) in GSE27555 had higher enrichment scores of the BRB up-regulated genes than non-responders (N) (*p* = 0.007). (**C**) There is no significant overlap between the differentially expressed genes in the comparison of BR vs BN and CR vs CN, as illustrated by Venn diagram. The major pathways up-regulated in CR and CN as well as the 2 up-regulated genes in common between the BR and CN groups are shown.

The results of functional analysis of the upregulated genes between BR and BN are reminiscent of the up-regulated genes in the BRB vs BRA comparison and suggest that many genes involved in HBV-related hepatic necroinflammation may also determine the response to IFNα treatment. We thus examined whether the 132 up-regulated genes in HBV inflammation (BRB vs. BRA) were also differentially expressed between BR vs BN. A total of 22 genes overlapped between these 2 datasets with statistical significance (Fig. 5A) (p = 9.63 × 10^−25^). This result was further confirmed by GSE27555. Like in the BR vs BN comparison, the enrichment scores of the 132 up-regulated genes of HBV inflammation in the responders of GSE27555 were also significantly higher than the scores in non-responders, indicating that the magnitude of host responses to the virus plays an important role in treatment response (Fig. 5B).

### The mechanisms responsible for IFNα treatment responses are different in HBV and HCV patients

IFNα is also used in the treatment of HCV infection. To study whether the host factors responsible for IFNα response are similar in chronically HBV- and HCV-infected patients, we used a publicly available dataset, GSE11190, which contains hepatic RNA expression profiles of 9 HCV responders (CR) and 6 non-responders (CN) before IFN treatment. Consistent with the results of previous studies, IFN-stimulated genes (ISGs), such as 2′-5′-oligoadenylate synthetase, and other antiviral defense-related genes were enriched in the CN but not the CR group^14^,^15^,^16^ (Fig. 5C). On the other hand, the acute inflammatory response, leukocyte migration, and response to cytokine stimulus were enriched in the CR group. There was no significant overlap between the up-regulated genes in either the BR and CR comparison or the BR and CN comparison (Fig. 5C). These analyses indicate that different underlying mechanisms are responsible for a favorable response to IFNα therapy in chronically HBV- and HCV-infected patients.

## Discussion

In this study, we characterized genes and pathways associated with CHB-associated necroinflammation by analyzing the expression profiles of paired liver biopsy samples of CHB patients before and after successful therapy with IFNα. Moreover, we examined the expression profiles of liver biopsy tissues from IFNα responders and non-responders to identify the genetic determinants of responsiveness of IFNα therapy. Our results revealed that immune-related genes and pathways are the dominant host responses to chronic active HBV infection and the major determinants of response to IFNα therapy. Furthermore, lower intrahepatic levels of HBV pregenomic RNA also favor IFNα treatment response. Although a relatively small set of patients were analyzed owing to the limitations of liver sample acquisition, the significant *p* values of a series of analyses and confirmation of our findings in independent clinical cohorts illustrated the reliability of our results.

To our knowledge, this is the first study using human paired liver biopsy samples to characterize the host responses to chronic HBV infection in terms of the transcriptome. Our study clearly showed that HBV continually evoked host immune responses even after long-term chronic infection, at least in the future IFNα responders. In line with this finding, GSEA analysis demonstrated that immune-related genes associated with chronic infection in the woodchuck model or viral clearance in the acute infection chimpanzee model were all enriched in the BRB samples. These results indicate that hosts respond to hepadnaviruses similarly no matter the infection is acute or chronic. In consistent with our analysis, Feltch *et al*. also found that the majority of genes associated with clearance of acute HBV infection in chimpanzees were also differentially expressed during self-limiting infection of WHV as well as in persistently infected woodchucks^17^. These results suggested that the outcome of infection may actually be determined by the magnitude of the overall immune response or the levels of certain key molecules but not by different transcriptional signatures. Alternatively, the genes commonly induced in acute and chronic HBV infection may not play major roles in eliminating the virus or their functions are impaired in chronic HBV infection.

In this study, we found that IFNγ rather than type I IFNs was the dominant IFN response in persistently HBV-infected individuals. A similar result was reported in the chronically WHV-infected woodchuck model^17^. IFNγ has been shown to be a critical mediator in noncytolytic control of acute HBV in a transgenic mice model^18^, however, these results revealed that hepadnavirus infection persists despite the presence of intrahepatic transcriptional response of IFNγ. Another notable finding is that B cell-associated genes were up-regulated significantly in BRB samples as well as in IFN responders, indicating that B cells are one of the dominant infiltrated immune cells in the CHB inflammatory livers and that B cell-related genes are important predictors of IFN treatment response. Unlike T cells, whose roles in determining the outcomes of HBV infection have been extensively investigated, the roles of B cells in CHB are less well characterized and may be overlooked. Our data highlighted the importance of B cells in inflammatory response and control of the virus in HBV patients. Further studies to unravel the underlying mechanisms for the lack of viral control by IFN-γ during persistent infection and the roles of B cells in viral control will have significant implications when developing immunotherapeutic strategies for chronic hepatitis B.

We also used intrahepatic gene expression profiling to explore the possible molecular basis for response to IFNα treatment in CHB patients. Previous studies have found that both viral and host factors influence the response to IFNα^9^,^19^,^20^. Among these factors, low serum HBV is generally considered to be one of the most reliable predictors of favorable response to IFNα treatment^19^,^20^. In this study, we found a significant association between higher intrahepatic pregenomic/precore RNA levels before treatment and non-responsiveness to IFNα therapy (Fig. 3A). The higher total HBV RNA also showed a trend toward association with IFNα treatment failure, though it was not statistically significant (Fig. 3B). Increasing evidence suggests that HBV has developed mechanisms to counteract the host innate immune responses through the viral encoded proteins^21^. Therefore, one possible explanation for the association of lower HBV expression/replication with favorable response to IFNα therapy is that the antiviral and immunomodulatory activities of IFNα may be attenuated by higher levels of viral proteins. These data thus emphasized the importance of lowering viral titers and/or expression levels before IFN therapy for a favorable response. Recent clinical studies have supported the notion that combining IFN with anti-viral therapy resulted in better therapeutic outcomes^22^,^23^.

We further used genome-wide exon arrays to study the host factors determining the treatment response to IFNα in CHB patients. We found that the immune responses before IFN therapy were more active in responders than in non-responders, as reflected by the higher expression level of MHC class II molecules, lymphocyte activation markers *(CD69 and CR2)* and other immune related genes as well (Fig. 4). Therefore, responders may represent those people who have better immune responses in nature to constrain the virus, another explanation for the association of lower intrahepatic viral pregenomic RNA levels with response to IFNα treatment. Exogenous administration of IFNα then further boosted the immune response to control the virus. It is thus not surprising that up-regulated genes in the microinflammatory livers (BRB) are highly enriched in the up-regulated genes of responders (Fig. 5A,B)^12^,^24^. HBsAg clearance is the most important end point of therapy and could be achieved in some IFN-α responders. Unfortunately, all the responders in this study only achieved ALT normalization, HBe seroconversion and decrease of viral load (<20,000 IU/ml) at the end of the post-treatment 6-month follow-up. We could not further analyze the genes associated with loss of HBsAg among these IFN-α responders.

We used the top 25 up-regulated genes in BR vs BN as a predictive gene signature to predict treatment response. ROC curve analyses of the data in GSE27555 and GSE54747 validated the predictive accuracy of this signature for IFN treatment response. Employing hepatic gene expression as a treatment response predictor is not practical in current clinical practice. However, the information contained in the livers could provide important clues to what we can focus on in the more accessible blood samples in the future, for example, the secretory proteins, such as cytokines/chemokines, or cell surface markers of immune cells.

IFNα has also been used to treat chronic HCV infection. Several studies suggested that baseline levels of ISGs before IFN-α treatment could predict the treatment response of HCV patients. Non-responders tended to have pre-elevated levels of intrahepatic ISGs but responders did not^25^,^26^. Surprisingly, our data as well as GSE54747^24^ could not find an association of higher baseline levels of ISGs with treatment failure in HBV patients. A recent study using the woodchuck model of CHB also revealed that baseline ISG expression was not an important determinant of IFN-α treatment response^27^. Comparing the up-regulated genes of HBV responders in our study with those of HCV responders or HCV non-responders in the dataset of GSE11190 did not find any significant overlap (Fig. 5C). These results suggested that the underlying mechanisms for successful IFN treatment are different in chronic HBV and HCV patients. The difference may be attributed to the intrinsic properties of these 2 viruses. The double-stranded RNA replicative intermediates of HCV can readily be sensed by innate immunity and thereby induce robust IFNα/β responses. In contrast, HBV’s replicating genome is sheltered within viral capsid particles in the cytoplasm and is relatively invisible to the innate immunity sensing machinery. Exogenous IFN offers little benefit for those HCV patients who have already achieved higher or maximal ISG expression before treatment. On the other hand, HBV responders may benefit from IFNα therapy by boosting the preexisting immune response to HBV, the major determinant of treatment response for CHB patients in our analysis.

One major concern of this study is that we used the RNA extracted from liver biopsy tissues which are composed of different cell types, including hepatocytes, nonparenchymal cells and infiltrated immune cells. Therefore, the gene expression profile we analyzed is the sum of all cell types in the biopsy sample. Some changes might be overshadowed by the nonreacting cells. Furthermore, the cell compositions of the liver can be affected by immune responses and thus differ greatly before and after IFN treatment or between responders and non-responders. It is thus difficult to judge whether the changes of gene expression were occurred in hepatocytes or other cells. Using sorted cells could provide a clearer picture. However, enough numbers of different cell types could hardly be purified from the small size liver biopsy samples for analysis. We used cell-type specific signatures to partially overcome this problem. Validation the changes of some genes identified by arrays in BRB vs BRA or in BR vs BR in hepatocyte-derived cells culture system can provide experimental data not only to support the conclusion of array findings but also offer evidence that the changes of these genes may come from hepatocytes. Although we did not do such experiments by ourselves, several genes identified in BRB vs BRA could be found also to be upregulated in hepatocyte derived cells with HBV when compared to their parental cells (similar to BRB vs BRA), including cell cycle-related genes (CDKN1A and CCND1^28^), IFN-induced genes (TAP1^29^, STAT1, WARs, GBP1, CXCL9, CXCL10, CXCL11^30^) and oncogenic miRNA21^31^,^31^.

In summary, our study revealed that the immune system continually responds to hepadnavirus during chronic infection. IFNγ and B cells are the major interferon and dominant immune cells, respectively, in the inflammatory livers of CHB patients. Both the baseline levels of HBV pregenomic RNA and host immune responses but not ISGs are key determinants of the response to IFNα treatment for HBV patients. In total, 25 host genes can be used as predictors of response to IFNα therapy. These findings provide important information on the host responses to CHB infection and novel insights into the personalized management of CHB patients with IFNα treatment in clinical settings.

## Additional Information

**How to cite this article**: Wu, H.-L. *et al*. Liver Gene Expression Profiles Correlate with Virus Infection and Response to Interferon Therapy in Chronic Hepatitis B Patients. *Sci. Rep*. **6**, 31349; doi: 10.1038/srep31349 (2016).

## Supplementary Material

Supplementary Information
