# Supplementary material for: Liver Gene Expression Profiles Correlate with Virus Infection and Response to Interferon Therapy in Chronic Hepatitis B Patients
Source: Sci Rep. 2016 Aug 22;6:31349. doi: 10.1038/srep31349 (PMC4992874; doi:10.1038/srep31349)
Supplement: Supplementary Information [file srep31349-s1.pdf]

Supplementary information to:

Liver Gene Expression Profiles Correlate with Virus Infection and Response to Interferon Therapy in Chronic Hepatitis B Patients

Hui-Lin Wu, Tzu-Hung Hsiao*, Pei-Jer Chen, Siao-Han Wong, Jia-Horng Kao, Ding-Shinn Chen, Jo-Yang Lu, Tzu-Pin Lu, Yidong Chen, Eric Y. Chuang, Hui-Chu Tu, and Chun-Jen Liu*

Supplemental materials and methods

Hepatitis virus markers and virologic assays

Serum specimens were tested for HBsAg, anti-HBs, HBeAg, anti-HBe, and anti-delta with commercially available kits (Ausab, Ausria II, Murex HBeAg/anti-HBe, and Anti-delta, Abbott Laboratories, North Chicago, IL, USA). Antibodies to the hepatitis c virus (HCV) were tested by a second-generation enzyme-linked immunoassay (Abbott Laboratories, North Chicago, IL, USA). Serum HBV DNA was determined by an in-house real-time PCR assay as described previously[1].

Histologic evaluations

Liver histology was scored by the Knodell system, which takes into account the severity of periportal, portal and lobular necroinflammation as well as fibrosis. Part of the liver biopsy specimen was stored in liquid nitrogen until further RNA assay.

Quantitative RT-PCR of the intrahepatic HBV transcripts

To determine the intrahepatic levels of HBV transcripts by qRT-PCR, 0.5 μg of purified total RNA was reverse-transcribed to cDNA by Moloney murine leukemia virus reverse transcriptase (M-MLV RT, Invitrogen Life Technologies, CA, USA) with oligo(dT) plus random hexamer primers according to the manufacturer’s protocol. Five μL of 10–fold diluted cDNA were used for qRT-PCR was amplified on a Roche LightCycler instrument with a LightCycler® FastStart DNA Master^{PLUS} SYBR Green I kit. Primer pairs HBV+1550F/HBV-1695R and HBV+1811F/HBV-2016R were used to detect total viral transcripts and precore/pregenomic RNA, respectively (Figure S6). The amplification conditions included initial denaturation at 95 ^{o}C for 10 s, followed by 40 cycles of denaturation at 95 ^{o}C for 10 s, annealing at 60 ^{o}C for 10 s and extension at 72 ^{o}C for 10 s. β-Actin was also amplified and used as an internal control for normalization. Copy numbers of HBV transcripts or actin mRNA were calculated by extrapolation from standard curves generated using 10-fold serial dilutions of plasmids containing known copies

of a monomeric genotype B HBV insert and/or a human β-actin fragment. The sequences of the PCR primers are specified in Table S7 and the locations of HBV primers in the HBV genome are depicted in Figure S6.

Gene expression dataset and data analysis

The raw data from the Affymetrix microarray was processed by the quantile and RMA algorithm. For the comparison of the BRB group vs the BRA group, Student's t-test was applied to estimate the statistical significance of the differences in gene expression. For the comparison of the BR and BN groups, the fold change of each gene was determined by calculating the difference between the averaged values of the probes on the gene.

Three datasets, GSE27555[2], GSE54747[3], and GSE11190[4], were downloaded from the gene expression omnibus (GEO) database[2]. GSE27555 and GSE54747 were used to validate the results derived from the BR vs BN comparison. GSE27555 contains expression profiles of 6 responders and 7 non-responders to IFNα treatment[2]; GSE54747 contains the profiles of 9 responders and 6 non-responders to IFNα and adefovir combined therapy[3]. The dataset GSE11190 was used for studying the IFN response in chronic HCV patients[5]. Only the profiles of liver biopsy samples before treatment were used in this study.

Gene set enrichment analysis

Three kinds of enrichment analysis were utilized for different scenarios. For the BRB vs BRA comparison, Gene Set Enrichment Analysis (GSEA), the software for which was downloaded from http://www.broad.mit.edu/gsea/, was performed to identify the functional enrichment or depletion[6]. The gene sets Biocarta pathway, Gene Ontology, and transcription factor targets (TFT), which were downloaded from MsigDB[6], were used for the analysis. The customized gene sets of white cells, interferon response, woodchuck, and chimpanzee were also generated and used to estimate the enrichment. Fisher's exact test was used to determine whether the overlap between 2 groups of differentially expressed genes reached statistical significance. The DAVID online analysis tool (website: http://david.abcc.ncifcrf.gov/)[7] was used to identify the enriched function of the differentially expressed genes.

Enrichment score

In order to estimate the enrichment level of gene sets for the expression of HBV infections and IFNα response, an enrichment scoring method based on the work of Tain *et al.*[8] was used. Suppose there are N genes in a given gene set. Let $\mathbf{x}_l = \{x_{1,l}, \ldots, x_{N,l}\}$,

where $x_{j,l}$ is the $\log_2$-transformed expression level of gene j in the expression profile l. For a given gene set, the enrichment score of the expression profile l is defined as

$$s_l = \frac{1}{N}\sum_{j=}^{N} z_{j,l} \tag{1}$$

where $z_{j,l} = (x_{j,l} - \mu_j)/\sigma_j$, μ_j is the mean of gene j and σ_j is the standard deviation of gene j in all expression profiles. To assess the statistical significance of the enrichment score s_l, we provide a permutation-based hypothesis test to estimate the statistical significance based on the concept proposed in Tain *et al.*[8]. The null distribution $Q1$ is generated by randomly selecting gene members of the gene set D times. Then the empirical p-values of the gene set are calculated as the fraction of the permutation values s_{Q1} that exceeds (or is below) the value s_l :

$$p_{Q1}(s_l) = \begin{cases} \#\{S_{Q1} > S_l\}/_D \text{ , enrichment} \\ \#\{S_{Q1} < S_l\}/_D \text{ , depletion} \end{cases} \tag{2}$$

Based on the enrichment score S_l and p-value, we can determine if the gene set under consideration is enriched (or depleted) in the expression profile l.

We also utilized another type of enrichment analysis which estimates the statistical significance of the gene overlap between gene sets and differentially expressed genes. First, two vectors, **b** and **p**, were constructed. For a given gene set, the indicator vector **b** contains information on whether or not a gene belongs to the gene set. This is expressed as $\mathbf{b} = (b_i)_M$, where M is the number of genes, and $b_i = 1$ when i^{th} gene is in the gene set, otherwise 0. The matrix $\mathbf{p} = (p_i)_M$ is also an indicator vector of differentially expressed genes, where $p_i = 1$ when the ith gene is differentially expressed in the comparison, otherwise 0. The enrichment is defined as a scoring function C of the two matrixes **b** and **p**.

$$ES = \mathrm{C}(\boldsymbol{b}, \boldsymbol{p}) \tag{3}$$

Here we use Fisher's exact test as the score function $\mathrm{C}(\mathbf{b}, \mathbf{p})$. Let $a = \sum_{i=1}^{M} b_i$ be the number of genes in the gene set, $b = \sum_{j=1}^{M} p_j$ be the number of differentially expressed genes, and $c = \sum_{k=1}^{M} b_k p_k$ be the number of overlapped genes between the gene set and the differentially expressed genes. The p-value of Fisher's exact test between the genome segment and the gene set can be calculated by

$$P(x > c) = \sum_{h=c}^{\infty} \frac{\binom{a}{h}\binom{M-a}{b-h}}{\binom{M}{b}} \tag{4}$$

Based on the p-values, we can determine if the gene set was enriched in the

differentially expressed genes.

Generation of the gene sets

The customized gene sets used in this study were listed at Supplemental Table S8. We describe the details of the gene set generation utilized in the study on this section. We specified the sources of data, original studies, and the process used to generate the gene sets in the following.

White cell gene set

Three white cell gene sets (B cell, NK cell, and T cell) were generated based on the expression profiles of white cells in Waddell S. *et al.* [9]. Each gene set was constructed by the associated genes according the Table S5 in the study. A total of 179, 161, and 210 genes were involved in the B cell, NK cell, and T cell gene sets, respectively.

Interferon response gene set

The interferon response gene sets included the response genes after IFNα and IFNγ treatment in primary human hepatocytes [10]. Three gene sets, IFNα, IFNγ, IFN$\alpha\cap\gamma$, were constructed based on the genes up–regulated in IFNα treated and IFNγ treated hepatocytes separately, and then in heptatocytes treated with either IFNα or IFNγ, according the Supplemental Table III in Dill *et al.* [10].

Woodchuck gene set

The woodchuck gene set was generated based on the dataset GSE36250 which contains the expression profiles of woodchuck tissues with WHV chronic infection in the GEO database[11]. The genes up-regulated in the profiles of chronically WHV-infected liver tissues compared with viral resolved tissues were collected as the gene set. Using fold change >4 and *p*-value <0.001 as the selection criteria, a total of 277 genes were collected in the gene set.

Chimpanzee gene set

The genes associated with viral clearance in the HBV-infected chimpanzees as recorded in the Table S3 of Wieland *et al.* [12] were collected as the chimpanzee gene set. The gene set contains 106 genes.

Microarray Sample Preparation, Hybridization, and Scanning

Frozen liver biopsy tissue specimens stored in liquid nitrogen were soaked in pre-chilled RNAlater-ICE (Ambion, Austin, Texas, USA) for at least 16 h before RNA

extraction. The biopsy tissues were homogenized in Trizol reagent (Invitrogen, Carlsbad, California, USA) using tissue grinders (Bioman scientific, Taipei, Taiwan) and then the total RNA was isolated and purified according to the manufacturer's instructions. RNA concentrations were measured by spectrophotometer. Before analysis of region-specific gene expression, the sensitivity and reliability of linear RNA amplification were examined. The quality and degradation of the isolated RNA were estimated after electrophoresis using an Agilent 2100 bioanalyzer (LabChip®, Agilent Technologies, Waldbronn, Germany). The isolated RNA was stored at -80°C until further analysis.

A total of 1 μg RNA was used for Affymetrix analysis. Microarray hybridization and scanning were performed following recommendations of the manufacturer. Briefly, Affymetrix microarrays (Affymetrix, Santa Clara, CA) were loaded with the fragmented target sample buffer mix. Each sample was individually hybridized for 16 h at 45 °C with rotation at 60 rpm. All of the washing and staining steps were performed using the Fluidics Station 450. The arrays were then scanned via the GeneChip Scanner 3000 (Affymetrix).

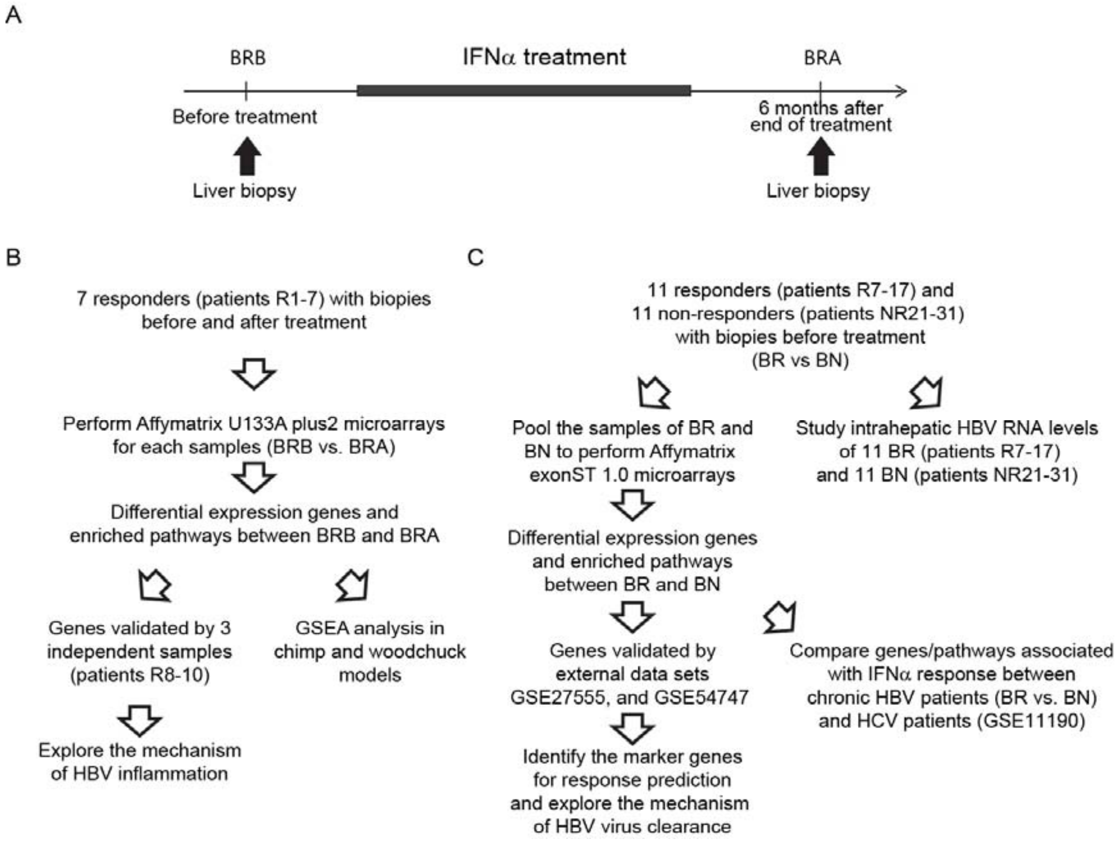

Figure S1. Analysis flow chart. Gene expression profiling of liver biopsies was performed to analyze the IFNα response in the inflammation of HBV infection. (A) The liver biopsies were taken before IFNα treatment and 6 months thereafter. (B) Seven pairs of samples before and after treatment were analyzed to identify the differential gene expression and enriched functions and pathways in the inflammation of HBV infection. The identified genes were validated in 3 independent samples. The gene expression profiles were also compared with the up-regulated genes in chimpanzee and woodchuck models through GSEA analysis. (C) Eleven samples each from responders and non-responders were pooled separately to perform Affymetrix EXON ST 1.0 array analysis, which contains more than 1 million probes. The genes predictive of the response to treatment and the pathways in which they are involved can be identified. The results were validated in two publicly available, independent data sets, GSE27555 and GSE54747.

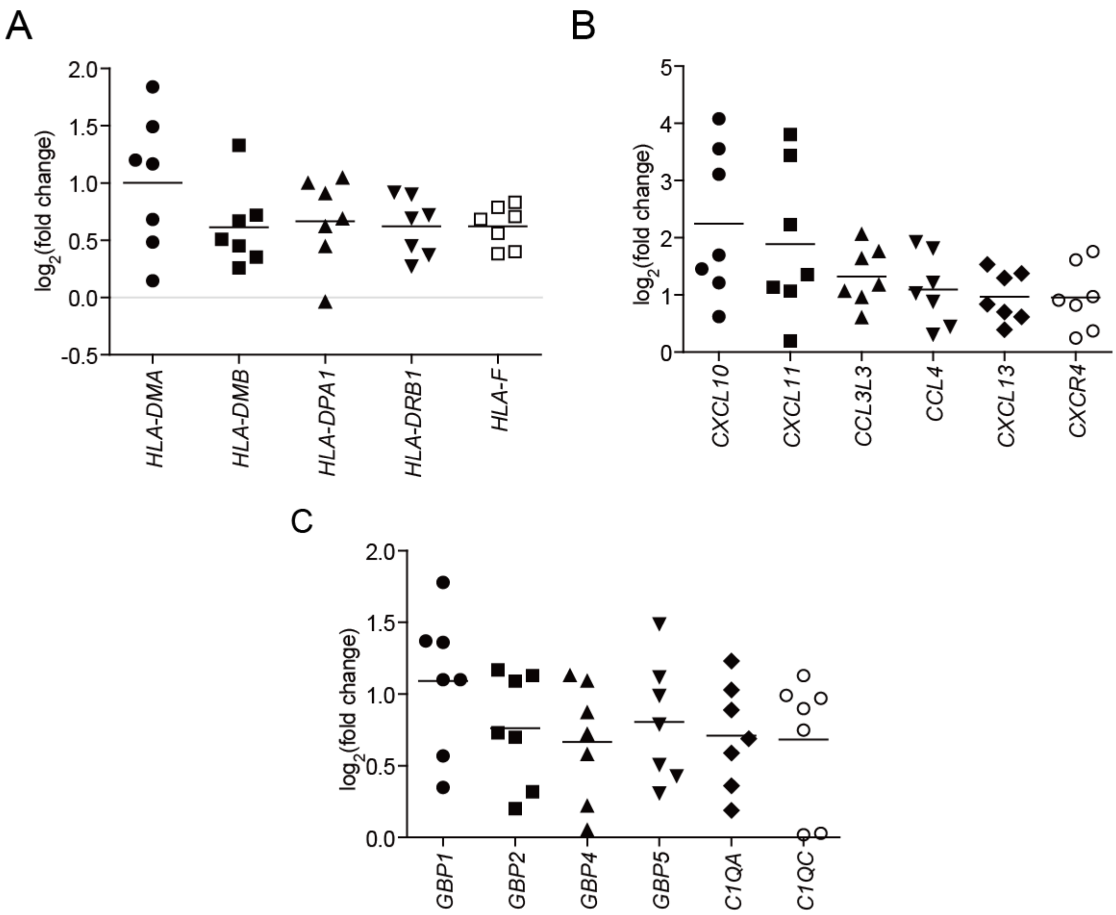

Figure S2. The representative up-regulated genes in the comparison of BRB vs BRA. HLA family genes (A), chemokines (B), GBP family genes, and complement related genes (C) were up-regulated in the comparison of BRB vs BRA.

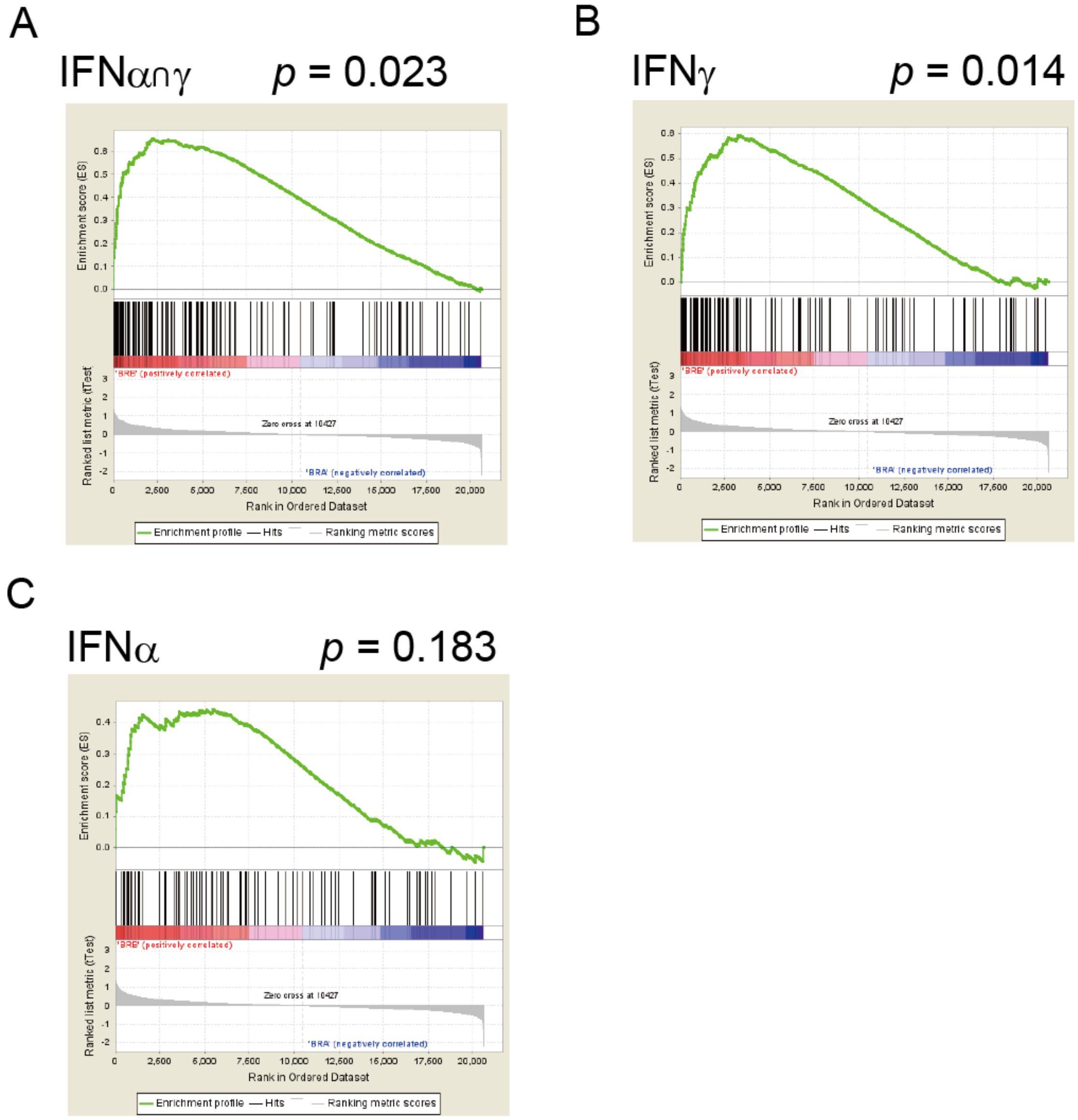

Figure S3. GSEA results of interferon signatures. Three interferon signatures, IFNα∩γ, IFNγ, and IFNα, were tested for enrichment in the BRB vs. BRA comparison. The IFNα∩γ gene set contains the genes commonly up-regulated in both the IFNγ– and IFNα-treated hepatocytes. The IFNγ and IFNα gene signatures contain genes specifically up-regulated in the IFNγ and IFNα treated hepatocytes, respectively. The GSEA results indicated the IFNα∩γ and IFNγ gene signatures were enriched ($p < 0.05$).

A

B cell

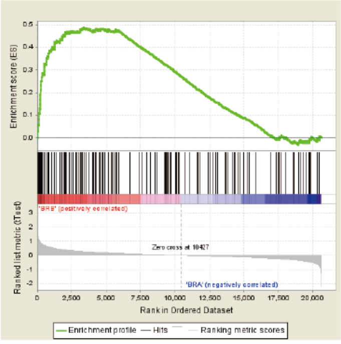

p = 0.049

B

NK cell

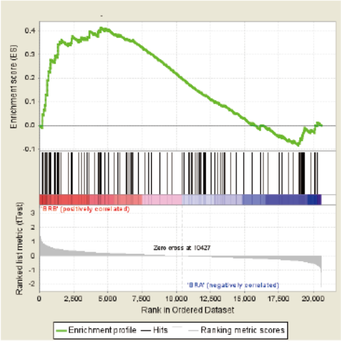

p = 0.113

C

T cell

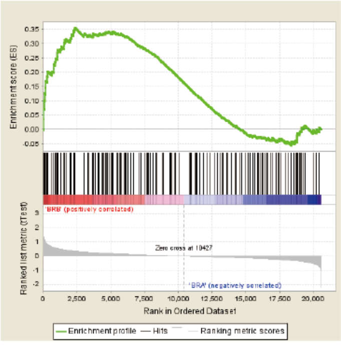

p = 0.204

Figure S4. GSEA results of leukocyte signatures. Three leukocyte signatures—B cell, NK cell, and T cell—were used to assess the signatures enriched in the BRB vs. BRA comparison. Only the B-cell gene signature was shown to have significant enrichment ($p < 0.05$).

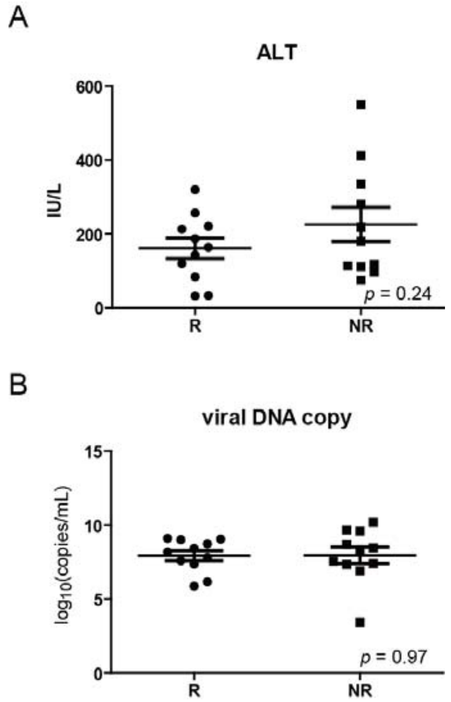

Figure S5. Serum levels of ALT and HBV DNA in IFN responders and non-responders with HBV. There was no significant difference in the serum levels of ALT and HBV DNA between responders (BR) and non-responders (BN).

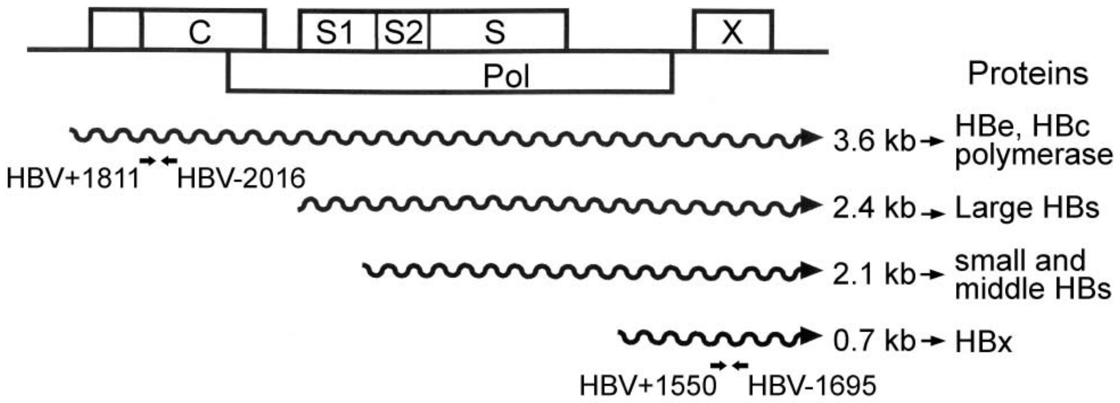

Figure S6. Schematic representation of HBV genome, including the four major transcripts and their protein products. The coding regions for HBV core, surface, polymerase, and X proteins are designated as C, S, Pol, and X, respectively. The relative locations of the PCR primers are also depicted. The primer pairs of HBV+1181/HBV-2016 and HBV+1550/HBV-1695 were used to determine the expression levels of the 3.6-kb precore/pregenomic RNA and total RNA, respectively.

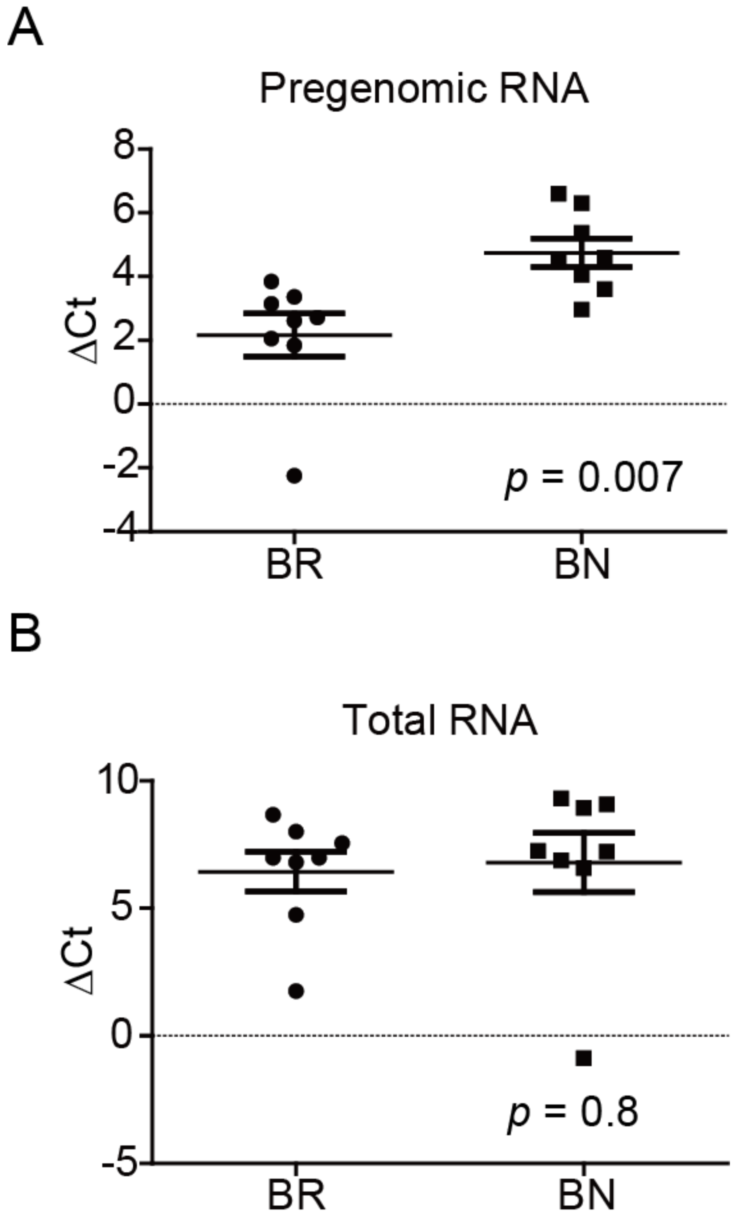

Figure S7. The expression levels of HBV pregenomic/precore RNA and total RNA in the validation cohort. Only pregenomic/precore RNA had differential expression between responders (BR) and non-responders (BN). The BN group had higher expression levels than the BR group.

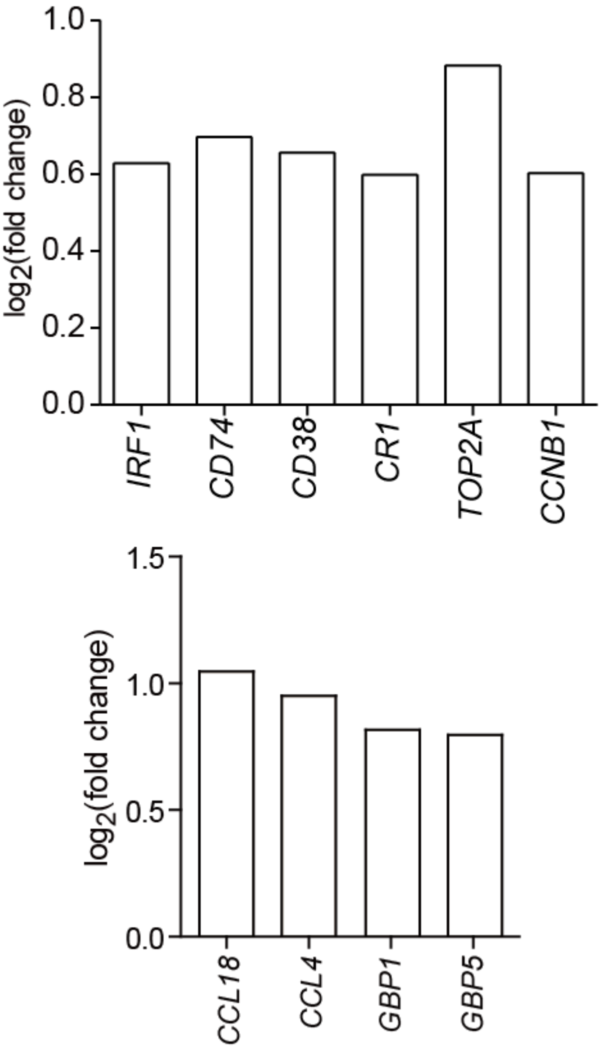

Figure S8. The relative expression levels of the immune- and proliferation-related genes in the comparison of BR vs BN. Immune-related genes**,** *IRF1*, CD antigens, chemokines, and GBPs were up-regulated in BR patients. *TOP2A* and *CCNB1*, which are associated with cell proliferation, were also up-regulated.

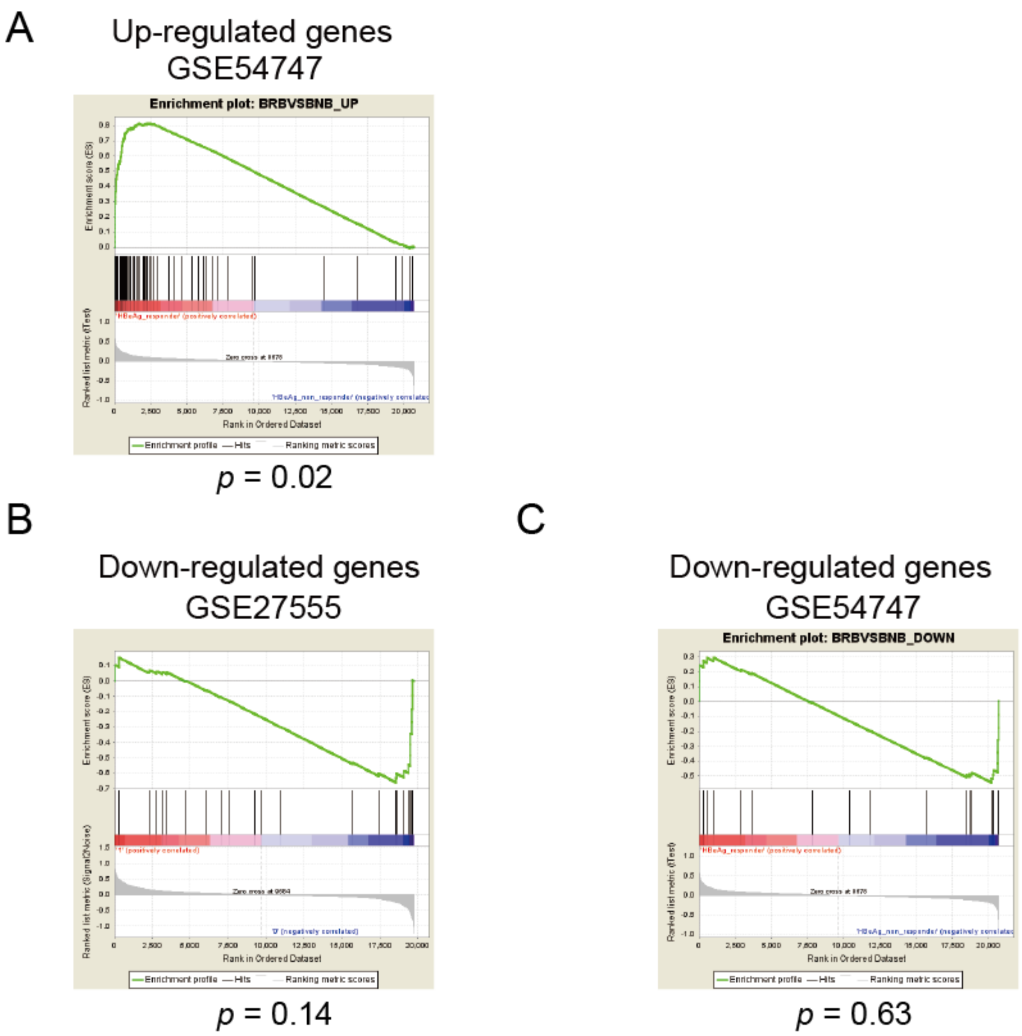

Figure S9. Validation of the up- and down-regulated genes identified in BR vs BN. A total of 118 up- and 33 down-regulated genes passed the selection criteria and were defined as up- and down-regulated differential expressed genes. The result of GSEA analysis showed that only the up regulated genes show the enrichment with statistical significance in the GSE54747 data sets. The down-regulated genes did not meet the significance criteria in the GSE27555 and GSE54747 datasets.

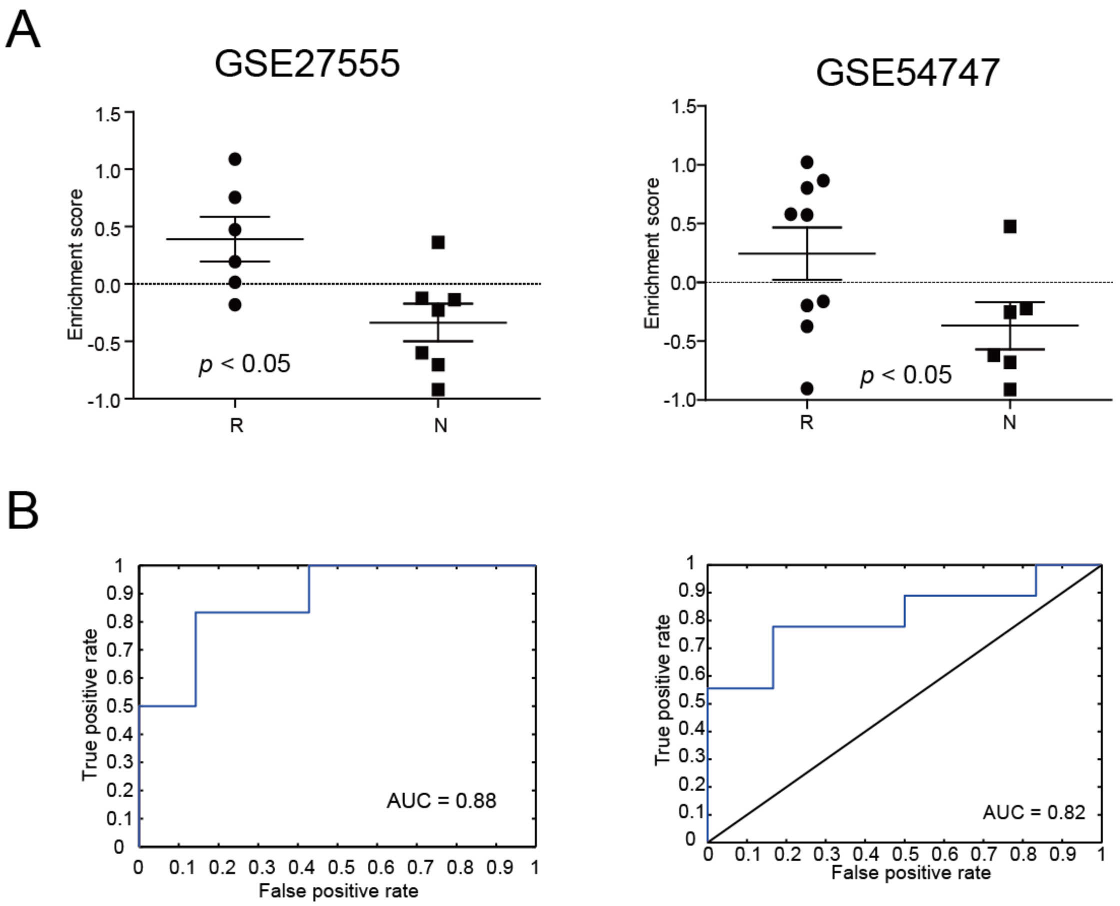

Figure S10. Validation of the up-regulated genes of BR vs BN in the GSE27555 and GSE54747 data set. (A) Utilizing the 118 up-regulated genes identified in the BR vs BN comparison as predictors, the enrichment scores of responders (R) were significantly higher than those of non-responders (N) ($p < 0.05$: Wilcoxon test) in GSE27555 and GSE54747 datasets. (B) The ROC analysis was performed. The AUC value was 0.88 and 0.82 in GSE27555 and GSE54747, respectively.

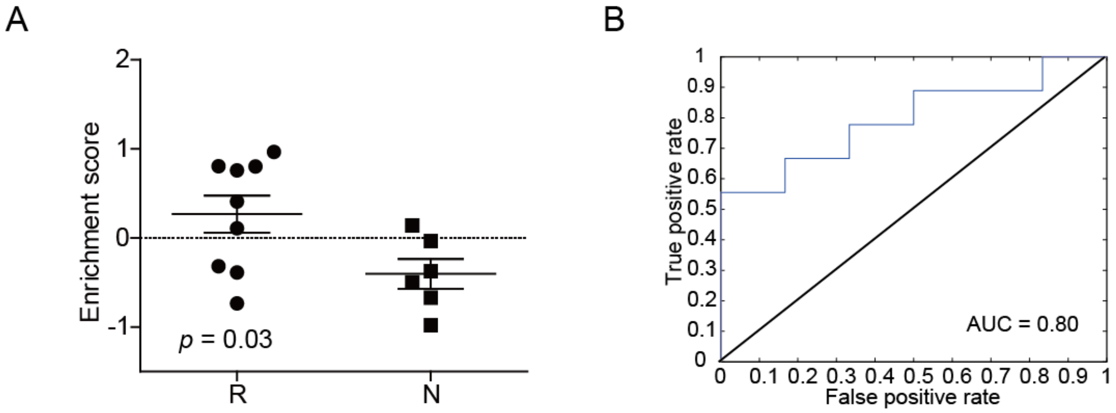

Figure S11. Validation of the 25 gene signature in the GSE54747 data set. (A) Utilizing the 25 gene signature as predictors, the enrichment scores of responders (R) were significantly higher than those of non-responders (N) (p = 0.03: Wilcoxon test). (B) The ROC analysis of the validation data sets, GSE547474, was performed. The AUC value was 0.80.

TableS 1. Characteristics of patients according to responses to interferon alpha-2b therapy

	**Responders N=19**	**Non-responders N=19**	***P* value**
**Baseline features**			
Gender			0.23
Male/female	13 (68.4) / 6 (31.6)	17 (89.5) / 2 (10.5)	
Age, year			0.31*
Mean ± SD	33.3 ± 12.2	34.7 ± 7.4	
Weight, kg			0.86 *
Mean ± SD	64.3 ± 11.3	65.2 ± 10.3	
Height, cm			0.92 *
Mean ± SD	167.2 ± 6.3	166.2 ± 6.1	
ALT level, IU/L			0.63 *
Mean ± SD	174.5 ± 82.4	216.7 ± 133.2	
Log(10) HBV DNA titer, copies/mL			0.6 *
Mean ± SD	8.66 ± 8.84	9.27 ± 9.59	0.1389 *
HBeAg positivity	18 (100%)	18 (100%)	1
HBV genotype			
B/C	10/9	9/10	0.837
Histology fibrosis stage (Metavir)			
F1/F2/F3/F4	3/7/5/4	5/5/5/4	0.628

*t-test. Values expressed as number (percentage) or mean ± standard deviation (SD).

Table S2. Patient information of the study cohort

Patient no	Gender	Age	ALT	Viral titer in serum	Response to IFN Tx	Study group in BRB vs BRA	Study group in BR vs BN	Study group in HBV expression in liver
R1	M	30	308	4.92E+07	R	testing (BRB1,RBA1)		validation
R2	M	22	143	3.09E+08	R	testing (BRB2,RBA2)		validation
R3	F	43	220	2.14E+08	R	testing (BRB3,RBA3)		validation
R4	M	32	99	3.83E+08	R	testing (BRB4,RBA4)		validation
R5	M	35	192	2.75E+09	R	testing (BRB5,RBA5)		validation
R6	M	27	276	5.04E+07	R	testing (BRB6,RBA6)		validation
R7	M	69	221	3.95E+07	R	testing (BRB7,RBA7)		testing
R8	F	21	84	6.34E+07	R	validation (BRB8)		testing
R9	F	28	187	1.02E+09	R	validation (BRB9)		testing
R10	F	25	257	1.12E+09	R	validation (BRB10)		testing
R11	M	35	32	1.52E+08	R			testing
R12	F	24	213	2.30E+07	R		BR	testing
R13	M	19	143	2.61E+08	R			testing
R14	M	28	33	1.50E+06	R			testing
R15	M	26	320	5.40E+08	R			testing
R16	M	35	120	1.23E+09	R			testing
R17	F	51	164	7.35E+05	R			testing
R18	M	35	173	9.77E+04	R			validation
R19	M	47	130	2.46E+06	R			validation
NR20	M	49	550	3.80E+09	NR			testing
NR21	F	33	98	2.14E+07	NR			testing
NR22	M	30	180	2.73E+03	NR			testing
NR23	M	36	112	4.92E+08	NR			testing
NR24	M	42	218	1.50E+10	NR			testing
NR25	M	42	335	3.60E+07	NR		BN	testing
NR26	M	33	117	2.90E+08	NR			testing
NR27	M	32	114	8.01E+06	NR			testing
NR28	M	34	280	2.49E+07	NR			testing
NR29	M	31	75	4.58E+09	NR			testing
NR30	M	32	412	2.06E+08	NR			testing
NR31	M	24	246	2.39E+06	NR			validation
NR32	M	23	130	7.93E+08	NR			validation
NR33	F	42	201	1.59E+09	NR			validation

NR34	M	38	369	1.27E+09	NR			validation
NR35	M	32	87	1.82E+08	NR			validation
NR36	M	49	109	2.74E+08	NR			validation
NR37	M	32	351	1.79E+08	NR			validation
NR38	M	26	133	1.17E+06	NR			validation
					38	10	24	38

TableS3.The 171 differentially expressed genes of the comparision of BRB vs. BRA

Probe	Symbol	log2(fold change)	p-value	Probe	Symbol	log2(fold change)	p-value
204533_at	CXCL10	2.25	0.0046	201289_at	CYR61	0.89	0.0106
211122_s_at	CXCL11	1.89	0.0182	210538_s_at	BIRC3	0.88	0.0169
202345_s_at	FABP5	1.81	0.0012	226474_at	NLRC5	0.88	0.0004
209969_s_at	STAT1	1.57	0.0007	205267_at	POU2AF1	0.87	0.006
205114_s_at	CCL3L3	1.32	0.0003	202307_s_at	TAP1	0.87	0.0009
222838_at	SLAMF7	1.21	0.0033	205831_at	CD2	0.85	0.0032
209395_at	CHI3L1	1.19	0.2785	201422_at	IFI30	0.85	0.0008
231956_at	RNF213	1.14	0.0011	203471_s_at	PLEK	0.83	0.0032
204103_at	CCL4	1.1	0.0005	204279_at	PSMB9	0.82	0.003
202269_x_at	GBP1	1.09	0.0023	207536_s_at	TNFRSF9	0.82	0.0002
219099_at	C12orf5	1.04	0.0167	202157_s_at	CELF2	0.81	0.001
214511_x_at	FCGR1B	1.01	0.0063	238581_at	GBP5	0.81	0.048
208949_s_at	LGALS3	1.01	0.0108	204446_s_at	ALOX5	0.8	0.0137
219386_s_at	SLAMF8	1.01	0.0018	202284_s_at	CDKN1A	0.8	0.0059
217478_s_at	HLA-DMA	1	0.0025	200629_at	WARS	0.8	0.0023
225973_at	TAP2	0.98	0.0088	239629_at	CFLAR	0.79	0.0058
205242_at	CXCL13	0.97	0.001	215193_x_at	HLA-DRB4	0.79	0.0018
201141_at	GPNMB	0.97	0.0118	224917_at	MIR21	0.79	0.0033
217028_at	CXCR4	0.96	0.0741	203409_at	DDB2	0.78	0.0021
1568592_at	TRIM69	0.96	0.0029	231776_at	EOMES	0.78	0.0008
213293_s_at	TRIM22	0.95	0.0013	200660_at	S100A11	0.78	0.0008
211762_s_at	KPNA2	0.94	0.0005	202897_at	SIRPA	0.77	0.0017
212998_x_at	LOC100133583	0.94	0.0295	231093_at	FCRL3	0.76	0.0016
205758_at	CD8A	0.92	0.0062	202748_at	GBP2	0.76	0.0014
225502_at	DOCK8	0.92	0.0064	209312_x_at	HLA-DRB5	0.76	0.0073
221087_s_at	APOL3	0.91	0.0192	227346_at	IKZF1	0.76	0.0159
209619_at	CD74	0.91	0.0006	205681_at	BCL2A1	0.75	0.0424
208998_at	UCP2	0.91	0.002	219505_at	CECR1	0.75	0.0169
224451_x_at	ARHGAP9	0.9	0.0009	208018_s_at	HCK	0.75	0.004
227677_at	JAK3	0.9	0	234987_at	SAMHD1	0.75	0.0214

Probe	Symbol	log2(fold change)	p-value	Probe	Symbol	log2(fold change)	p-value
203761_at	SLA	0.75	0	202800_at	SLC1A3	0.68	0.0028
223502_s_at	TNFSF13B	0.75	0.014	235175_at	GBP4	0.67	0.0085
229390_at	FAM26F	0.74	0.0171	213537_at	HLA-DPA1	0.67	0.057
226525_at	STK17B	0.74	0.0224	202803_s_at	ITGB2	0.67	0.0008
213193_x_at	TRBC1	0.74	0.0024	218237_s_at	SLC38A1	0.67	0.0876
204639_at	ADA	0.73	0.0017	208478_s_at	BAX	0.66	0.0001
226184_at	FMNL2	0.73	0.0006	229723_at	TAGAP	0.66	0.0107
206247_at	MICB	0.73	0.0007	202510_s_at	TNFAIP2	0.66	0.0028
209670_at	TRAC	0.73	0.0102	204122_at	TYROBP	0.66	0.0257
242234_at	XAF1	0.73	0.0004	208711_s_at	CCND1	0.65	0.0453
212063_at	CD44	0.72	0.0208	211776_s_at	EPB41L3	0.65	0.0064
203416_at	CD53	0.72	0.0057	219519_s_at	SIGLEC1	0.65	0.002
226219_at	ARHGAP30	0.71	0.0064	202391_at	BASP1	0.64	0.003
218232_at	C1QA	0.71	0.0091	202957_at	HCLS1	0.64	0.0066
205270_s_at	LCP2	0.71	0.0017	222872_x_at	NABP1	0.64	0.0002
227609_at	EPSTI1	0.7	0.0163	209813_x_at	TARP	0.64	0.022
238725_at	IRF1	0.7	0.0034	217733_s_at	TMSB10	0.64	0.0002
203528_at	SEMA4D	0.7	0.0062	204205_at	APOBEC3G	0.63	0.0769
206034_at	SERPINB8	0.7	0.0242	228532_at	C1orf162	0.63	0.0018
201859_at	SRGN	0.7	0.0366	204070_at	RARRES3	0.63	0.0001
204661_at	CD52	0.69	0.0074	242146_at	SNRPA1	0.63	0.0201
202746_at	ITM2A	0.69	0.1017	226783_at	AGXT2L2	0.62	0.0122
200600_at	MSN	0.69	0.0032	221653_x_at	APOL2	0.62	0.0031
236293_at	RHOH	0.69	0.0028	218673_s_at	ATG7	0.62	0.0001
225353_s_at	C1QC	0.68	0.0141	208306_x_at	HLA-DRB1	0.62	0.0021
209606_at	CYTIP	0.68	0.051	221978_at	HLA-F	0.62	0.0015
221860_at	HNRNPL	0.68	0.0206	201720_s_at	LAPTM5	0.62	0.0215
210029_at	IDO1	0.68	0.0102	218165_at	MEAF6	0.62	0.0064
209626_s_at	OSBPL3	0.68	0.0006	225520_at	MTHFD1L	0.62	0.0023
218223_s_at	PLEKHO1	0.68	0.0011	213603_s_at	RAC2	0.62	0.0049

Probe	Symbol	log2(fold change)	p-value	Probe	Symbol	log2(fold change)	p-value
211072_x_at	TUBA1B	0.62	0.0004	205960_at	PDK4	-0.67	0.0006
204141_at	TUBB2A	0.62	0.0455	209735_at	ABCG2	-0.68	0.0003
201649_at	UBE2L6	0.62	0.0007	1553679_s_at	VKORC1L1	-0.68	0.0031
235927_at	XPO1	0.62	0.0059	1561101_at	JAKMIP2-AS1	-0.69	0.0071
230391_at	CD84	0.61	0.0198	205651_x_at	RAPGEF4	-0.69	0.0162
225971_at	DDHD1	0.61	0.0016	233059_at	KCNJ3	-0.7	0.0105
1556820_a_at	DLEU2	0.61	0.0233	232158_x_at	NIPAL1	-0.71	0.0304
203932_at	HLA-DMB	0.61	0.0067	205942_s_at	ACSM3	-0.72	0.0065
213915_at	NKG7	0.6	0.0145	208561_at	ABCC9	-0.76	0.0289
209785_s_at	PLA2G4C	0.6	0.1241	224797_at	ARRDC3	-0.76	0.0194
205821_at	KLRK1	0.59	0.0056	223721_s_at	DNAJC12	-0.76	0.013
222218_s_at	PILRA	0.59	0.0154	219732_at	LPPR1	-0.76	0.0191
211599_x_at	MET	-0.59	0.0171	219800_s_at	THNSL1	-0.79	0.005
211681_s_at	PDLIM5	-0.59	0.0057	1555612_s_at	G6PC	-0.81	0.0235
222634_s_at	TBL1XR1	-0.59	0.0221	1558010_s_at	SLC1A2	-0.84	0.031
209743_s_at	ITCH	-0.6	0	210738_s_at	SLC4A4	-0.85	0.0036
226225_at	MCC	-0.6	0.0132	1558028_x_at	LINC00657	-0.9	0.0029
211419_s_at	CHN2	-0.61	0.0001	210328_at	GNMT	-0.95	0.0312
1556009_at	PEX13	-0.61	0.0159	207626_s_at	SLC7A2	-0.98	0.0199
221881_s_at	CLIC4	-0.62	0.0018	222071_s_at	SLCO4C1	-1.02	0.0233
222939_s_at	SLC16A10	-0.62	0.0112	215506_s_at	DIRAS3	-1.26	0.0002
204120_s_at	ADK	-0.63	0.223				
213409_s_at	RHEB	-0.63	0.0391				
202234_s_at	SLC16A1	-0.63	0.0979				
208608_s_at	SNTB1	-0.63	0.0065				
1558722_at	ZNF252P	-0.64	0.0031				
223879_s_at	OXR1	-0.65	0.0217				
205363_at	BBOX1	-0.66	0.0337				
227526_at	CDON	-0.66	0.0001				
219313_at	GRAMD1C	-0.66	0.0118				

TableS4. The GSEA result : The enriched gene sets of BRB samples in the comparison of BRB vs. BRA

NAME	SIZE	ES	NES	NOM p-val	FDR q-val	FWER p-val	RANK AT MAX
V$NFKAPPAB65_01	188	0.44	1.60	0.0020	0.1539	0.3780	4479
V$CREL_01	204	0.46	1.64	0.0020	0.1645	0.2900	4219
V$NFKB_Q6	198	0.41	1.63	0.0020	0.1548	0.3000	3036
V$STAT1_03	183	0.29	1.40	0.0021	0.4536	0.8300	4543
V$NFKB_C	209	0.39	1.62	0.0040	0.1461	0.3410	4488
V$NFKAPPAB_01	191	0.44	1.60	0.0040	0.1444	0.3820	3110
GGGNNTTTCC_V$NFKB_Q6_01	102	0.51	1.65	0.0059	0.3888	0.2530	4018
V$PU1_Q6	175	0.43	1.67	0.0061	0.6272	0.2160	5204
V$NFKB_Q6_01	172	0.40	1.60	0.0078	0.1460	0.3940	3959
BIOCARTA_CASPASE_PATHWAY	22	0.69	1.73	0.0000	0.1012	0.2030	4255
BIOCARTA_NKCELLS_PATHWAY	18	0.71	1.72	0.0020	0.0909	0.2200	1862
BIOCARTA_CTLA4_PATHWAY	20	0.75	1.81	0.0020	0.1508	0.0890	913
BIOCARTA_IL10_PATHWAY	17	0.74	1.73	0.0040	0.1259	0.1950	4174
BIOCARTA_ERK_PATHWAY	27	0.52	1.74	0.0041	0.1746	0.1800	7486
BIOCARTA_HIVNEF_PATHWAY	55	0.53	1.66	0.0080	0.1319	0.3170	5074
BIOCARTA_IL2_PATHWAY	22	0.63	1.71	0.0080	0.0861	0.2360	3163
BIOCARTA_TPO_PATHWAY	23	0.50	1.57	0.0100	0.1532	0.5000	3955
INTERFERON-GAMMA-MEDIATED SIGNALING PATHWAY	63	0.77	1.80	0.0000	0.9838	0.3710	2635
CYTOKINE-MEDIATED SIGNALING PATHWAY	160	0.65	1.79	0.0000	0.5129	0.3790	3175
TYPE I INTERFERON-MEDIATED SIGNALING PATHWAY	62	0.67	1.75	0.0000	0.5431	0.5150	2457
T CELL DIFFERENTIATION	26	0.72	1.71	0.0000	0.3417	0.6760	3135
RESPONSE TO VIRUS	122	0.55	1.68	0.0020	0.2617	0.7540	3316
ODONTOGENESIS	31	0.54	1.48	0.0039	0.3325	0.9980	1963
NEGATIVE REGULATION OF TRANSFORMING GROWTH FACTOR BETA RECEPTOR SIGNALING PATHWAY	29	0.60	1.71	0.0039	0.4230	0.6520	3087
T CELL HOMEOSTASIS	20	0.69	1.65	0.0039	0.2521	0.8770	3163
AMINO ACID TRANSPORT	35	0.48	1.51	0.0040	0.3210	0.9960	1191

CELLULAR RESPONSE TO TUMOR NECROSIS FACTOR	17	0.82	1.49	0.0040	0.3217	0.9970	1286
POSITIVE REGULATION OF T CELL ACTIVATION	19	0.77	1.61	0.0041	0.2763	0.9410	903
REGULATION OF RHO PROTEIN SIGNAL TRANSDUCTION	60	0.43	1.71	0.0058	0.3716	0.6640	4007
RAS PROTEIN SIGNAL TRANSDUCTION	67	0.50	1.64	0.0058	0.2348	0.9050	4385
HETEROPHILIC CELL-CELL ADHESION	18	0.66	1.70	0.0061	0.2900	0.7180	762
POSITIVE REGULATION OF NITRIC OXIDE BIOSYNTHETIC PROCESS	27	0.65	1.67	0.0085	0.2475	0.8240	4352
RESPONSE TO PROGESTERONE STIMULUS	27	0.68	1.53	0.0099	0.3262	0.9950	3051

TableS5.The differentially expressed genes of the comparison of BR vs. BN

Symbol	log2(fold change)	Symbol	log2(fold change)	Symbol	log2(fold change)	Symbol	log2(fold change)	Symbol	log2(fold change)
HLA-DRB1	2.63	ANKRD22	0.82	GAPT	0.69	E2F8	0.63	NCRNA00189	-0.62
ASCL1	2.31	OR2L8	0.81	SQLE	0.68	IRF1	0.63	C5orf23	-0.62
HLA-DRB5	1.87	PLTP	0.81	ARHGAP11A	0.68	XIAP	0.62	OR10H2	-0.62
CD69	1.22	GBP5	0.80	MSR1	0.68	ZNF562	0.62	NOG	-0.63
CR2	1.18	MKI67	0.79	PLA2G7	0.68	SCAMP5	0.62	CDHR2	-0.63
ZNF675	1.11	SH2D1A	0.78	RMI1	0.67	HKDC1	0.62	C9orf31	-0.64
HIST1H2BM	1.07	THBS1	0.78	CST2	0.67	OGFRL1	0.61	CRYGD	-0.67
UPP2	1.06	GPR18	0.77	LILRB1	0.67	UCP2	0.61	USP9Y	-0.69
CCL18	1.05	CD27	0.77	APOL3	0.66	RAP1B	0.61	FMR1NB	-0.70
PGK2	1.04	RGS1	0.76	IGK@	0.66	ADRBK2	0.61	UTY	-0.71
NTS	1.02	C10orf18	0.76	LACTB2	0.66	TRAF1	0.61	C19orf59	-0.72
ICOS	1.01	TNFRSF9	0.76	ZFX	0.66	ANLN	0.61	ACTA1	-0.73
LTF	0.99	ZNF382	0.75	CD38	0.66	ANKRD36BP1	0.61	S100A7A	-0.74
CD274	0.96	PSMB9	0.74	CMKLR1	0.65	NCAPG	0.61	DDX3Y	-0.74
CCL4	0.95	SKAP1	0.73	ADAM6	0.65	CCNB1	0.60	EIF1AY	-0.75
CXCL9	0.94	FAM111B	0.72	RAD51AP1	0.65	TAP1	0.60	UNC93A	-0.75
CXCL10	0.94	RRM2	0.72	SCFD1	0.65	CR1	0.60	XPNPEP2	-0.77
IL2RG	0.93	CECR1	0.72	SUCNR1	0.65	CD68	0.60	GSTTP1	-0.77
C21orf91	0.92	SLC7A7	0.72	CINP	0.64	PRC1	0.60	PRAMEF1	-0.78
ARHGDIB	0.90	SIRPG	0.71	AKAP5	0.64	LILRB4	0.59	CETN3	-0.79
LGALS13	0.90	MRPS6	0.71	MCTP2	0.64	ZNF600	0.59	MYL1	-0.79
TOP2A	0.88	TNFSF13B	0.71	P2RY10	0.64	ZNF770	0.59	CKM	-0.80
STS	0.88	SAMHD1	0.71	ICAM1	0.64	ITK	0.59	CPE	-0.80
XCL1	0.88	XRCC2	0.71	ITM2A	0.64	ASPM	0.59	CYorf15B	-0.81
BIRC3	0.86	RAB31	0.70	CISH	0.64	FCGR1A	0.59	RPS4Y1	-0.88
SLAMF7	0.85	WARS	0.70	OSTalpha	0.64	KIF23	0.59	MYH2	-0.96
SIRPB1	0.83	CD74	0.70	TUBA1A	0.63	NFE2	0.59	MYL2	-1.01
KL	0.83	CCL26	0.69	KPNA2	0.63	SYK	0.59	SERPINA12	-1.02

ERV3	0.82	HLA-DMB	0.69	MIR650	0.63	LOC286359	-0.59	AKR1B15	-1.02
GBP1	0.82	ZNF266	0.69	FYB	0.63	GCNT4	-0.60	GPC3	-1.04
								MYBPC1	-1.20

TableS6. The enriched functional cluster of up-regulated genes in the comparison of BR vs. BN

Cluster 1	Enrichment Score: 3.33				
Category	Term	Count	%	PValue	Genes
GOTERM_BP_FAT	GO:0002684~positive regulation of immune system process	13	1.37	1.78E-07	ICAM1, CR1, CR2, SKAP1, CD74, CD38, SIRPG, SH2D1A, TNFSF13B, IL2RG, THBS1, CD27, SYK
GOTERM_BP_FAT	GO:0048584~positive regulation of response to stimulus	8	0.84	0.002	CR1, SH2D1A, CR2, TNFSF13B, THBS1, SKAP1, CD27, SYK
GOTERM_BP_FAT	GO:0050778~positive regulation of immune response	6	0.63	0.004	CR1, SH2D1A, CR2, TNFSF13B, SKAP1, SYK
Cluster 2	Enrichment Score: 2.91				
Category	Term	Count	%	PValue	Genes
GOTERM_BP_FAT	GO:0006952~defense response	19	2.00	5.03E-07	ITK, CR1, CR2, KL, CXCL9, SAMHD1, SLAMF7, CCL4, CD74, CCL18, CCL26, CXCL10, APOL3, SH2D1A, FCGR1A, TAP1, PLA2G7, LTF, THBS1
INTERPRO	IPR001811:Small chemokine, interleukin-8-like	6	0.63	6.49E-06	CXCL9, XCL1, CCL4, CCL18, CCL26, CXCL10
SMART	SM00199:SCY	6	0.63	1.15E-05	CXCL9, XCL1, CCL4, CCL18, CCL26, CXCL10
GOTERM_MF_FAT	GO:0008009~chemokine activity	6	0.63	1.33E-05	CXCL9, XCL1, CCL4, CCL18, CCL26, CXCL10
GOTERM_MF_FAT	GO:0042379~chemokine receptor binding	6	0.63	1.82E-05	CXCL9, XCL1, CCL4, CCL18, CCL26, CXCL10
SP_PIR_KEYWORDS	cytokine	8	0.84	1.03E-04	TNFSF13B, CXCL9, XCL1, CCL4, CISH, CCL18, CCL26, CXCL10
GOTERM_BP_FAT	GO:0006954~inflammatory response	11	1.16	1.42E-04	APOL3, CR1, CR2, KL, PLA2G7, CXCL9, THBS1, CCL4, CCL18, CCL26, CXCL10
GOTERM_BP_FAT	GO:0042330~taxis	8	0.84	1.81E-04	CMKLR1, CXCL9, XCL1, CCL4, CCL18, CCL26, CXCL10, SYK
GOTERM_BP_FAT	GO:0006935~chemotaxis	8	0.84	1.81E-04	CMKLR1, CXCL9, XCL1, CCL4, CCL18, CCL26, CXCL10, SYK
KEGG_PATHWAY	hsa04062:Chemokine signaling pathway	9	0.95	3.56E-04	ITK, CXCL9, ADRBK2, RAP1B, XCL1, CCL4, CCL18, CCL26, CXCL10
KEGG_PATHWAY	hsa04060:Cytokine-cytokine receptor interaction	10	1.05	7.44E-04	TNFRSF9, TNFSF13B, CXCL9, IL2RG, XCL1, CCL4, CD27, CCL18, CCL26, CXCL10
SP_PIR_KEYWORDS	chemotaxis	5	0.53	8.83E-04	XCL1, CCL4, CCL18, CCL26, CXCL10
SP_PIR_KEYWORDS	inflammatory response	5	0.53	0.001	CXCL9, CCL4, CCL18, CCL26, CXCL10
GOTERM_BP_FAT	GO:0009611~response to wounding	12	1.26	0.002	CCNB1, APOL3, CR1, CR2, KL, PLA2G7, CXCL9, THBS1, CCL4, CCL18, CCL26, CXCL10

GOTERM_MF_FAT	GO:0005125~cytokine activity	7	0.74	0.002	TNFSF13B, CXCL9, XCL1, CCL4, CCL18, CCL26, CXCL10
GOTERM_CC_FAT	GO:0005615~extracellular space	13	1.37	0.003	ICAM1, MSR1, KL, CXCL9, CECR1, CCL4, CCL18, CXCL10, CCL26, TNFSF13B, PLA2G7, XCL1, THBS1
GOTERM_BP_FAT	GO:0007626~locomotory behavior	8	0.84	0.004	CMKLR1, CXCL9, XCL1, CCL4, CCL18, CCL26, CXCL10, SYK
INTERPRO	IPR000827:Small chemokine, C-C group, conserved site	3	0.32	0.011	CCL4, CCL18, CCL26
Cluster 3	Enrichment Score: 2.72				
Category	Term	Count	%	PValue	Genes
GOTERM_BP_FAT	GO:0002252~immune effector process	8	0.84	5.95E-05	ICAM1, CR1, CR2, TNFSF13B, SAMHD1, SLAMF7, CD74, CD27
GOTERM_BP_FAT	GO:0002449~lymphocyte mediated immunity	6	0.63	1.65E-04	ICAM1, CR1, CR2, SLAMF7, CD74, CD27
**BIOCARTA**	**h_blymphocytePathway:B Lymphocyte Cell Surface Molecules**	**4**	**0.42**	**4.12E-04**	**ICAM1, CR1, CR2, HLA-DRB1**
GOTERM_BP_FAT	GO:0002443~leukocyte mediated immunity	6	0.63	4.33E-04	ICAM1, CR1, CR2, SLAMF7, CD74, CD27
GOTERM_BP_FAT	GO:0002460~adaptive immune response based on somatic recombination of immune receptors built from immunoglobulin superfamily domains	5	0.53	0.003	ICAM1, CR1, CR2, CD74, CD27
GOTERM_BP_FAT	GO:0002250~adaptive immune response	5	0.53	0.003	ICAM1, CR1, CR2, CD74, CD27
GOTERM_BP_FAT	GO:0016064~immunoglobulin mediated immune response	4	0.42	0.007	CR1, CR2, CD74, CD27
GOTERM_BP_FAT	GO:0019724~B cell mediated immunity	4	0.42	0.008	CR1, CR2, CD74, CD27
Cluster 4	Enrichment Score: 2.67				
Category	Term	Count	%	PValue	Genes
SP_PIR_KEYWORDS	disulfide bond	41	4.32	1.18E-07	OR2L8, MSR1, HLA-DRB1, CXCL9, CCL4, CD74, SIRPB1, CXCL10, SLC7A7, CCL26, CD68, CD69, ICOS, FCGR1A, HLA-DRB5, LTF,

					IL2RG, SUCNR1, THBS1, ERV3, CD27, PLTP, ICAM1, STS, CR1, CR2, GPR18, CMKLR1, CST2, LGALS13, SLAMF7, CCL18, LILRB1, TNFRSF9, CD38, SIRPG, P2RY10, TNFSF13B, LILRB4, CD274, XCL1
UP_SEQ_FEATURE	disulfide bond	38	4.00	1.44E-06	OR2L8, MSR1, HLA-DRB1, CXCL9, CCL4, CD74, SIRPB1, CXCL10, CCL26, CD68, CD69, ICOS, FCGR1A, HLA-DRB5, LTF, IL2RG, SUCNR1, THBS1, CD27, PLTP, ICAM1, STS, CR1, CR2, GPR18, CMKLR1, CST2, SLAMF7, CCL18, LILRB1, TNFRSF9, CD38, SIRPG, P2RY10, TNFSF13B, LILRB4, CD274, XCL1
GOTERM_CC_FAT	GO:0005886~plasma membrane	47	4.95	1.99E-05	OR2L8, MSR1, HLA-DRB1, HLA-DMB, SKAP1, CD74, SIRPB1, SLC7A7, ITM2A, CD68, CD69, ICOS, FCGR1A, TAP1, HLA-DRB5, IL2RG, SUCNR1, OSTALPHA, THBS1, CD27, SCAMP5, SYK, GAPT, ICAM1, ITK, STS, CR1, CR2, GBP5, GPR18, KL, CMKLR1, CISH, LILRB1, TNFRSF9, CD38, RAB31, SIRPG, SCFD1, P2RY10, RGS1, TNFSF13B, LILRB4, CD274, AKAP5, RAP1B, GBP1
SP_PIR_KEYWORDS	transmembrane protein	14	1.47	1.11E-04	STS, CR1, CR2, MSR1, HLA-DRB1, CD74, CD38, TNFRSF9, CD68, CD69, FCGR1A, HLA-DRB5, IL2RG, CD27
SP_PIR_KEYWORDS	signal	34	3.58	9.32E-04	HLA-DRB1, CXCL9, HLA-DMB, CCL4, SIRPB1, CXCL10, CCL26, CD68, FCGR1A, ICOS, LTF, HLA-DRB5, IL2RG, THBS1, ERV3, CD27, PLTP, ICAM1, CR1, STS, CR2, KL, CECR1, CST2, SLAMF7, CCL18, LILRB1, TNFRSF9, SIRPG, NTS, LILRB4, CD274, PLA2G7, XCL1
UP_SEQ_FEATURE	signal peptide	34	3.58	0.001	HLA-DRB1, CXCL9, HLA-DMB, CCL4, SIRPB1, CXCL10, CCL26, CD68, FCGR1A, ICOS, LTF, HLA-DRB5, IL2RG, THBS1, ERV3, CD27, PLTP, ICAM1, CR1, STS, CR2, KL, CECR1, CST2, SLAMF7, CCL18, LILRB1, TNFRSF9, SIRPG, NTS, LILRB4, CD274, PLA2G7, XCL1
UP_SEQ_FEATURE	topological domain:Extracellular	29	3.05	0.002	OR2L8, MSR1, HLA-DRB1, SIRPB1, CD74, CD68, CD69, FCGR1A, ICOS, HLA-DRB5, SUCNR1, IL2RG, CD27, SCAMP5, ICAM1, CR1, CR2, GPR18, CMKLR1, KL, SLAMF7, LILRB1, TNFRSF9, CD38, P2RY10, SIRPG, TNFSF13B, LILRB4, CD274
SP_PIR_KEYWORDS	receptor	20	2.11	0.002	TRAF1, OR2L8, CR1, CR2, MSR1, GPR18, CMKLR1, SLAMF7, OGFRL1, LILRB1, TNFRSF9, CD38, P2RY10, CD69, FCGR1A, LILRB4, CD274, IL2RG, SUCNR1, CD27
UP_SEQ_FEATURE	topological domain:Cytoplasmic	32	3.37	0.007	OR2L8, MSR1, HLA-DRB1, HLA-DMB, CD74, SIRPB1, CD68, CD69, FCGR1A, ICOS, TAP1, HLA-DRB5, IL2RG, SUCNR1, CD27, SCAMP5, ICAM1, CR1, STS, CR2, GPR18, CMKLR1, KL, SLAMF7, LILRB1, TNFRSF9, CD38, SIRPG, P2RY10, TNFSF13B, LILRB4, CD274
UP_SEQ_FEATURE	glycosylation site:N-linked (GlcNAc...)	37	3.89	0.008	OR2L8, MSR1, HLA-DRB1, HLA-DMB, CD74, SIRPB1, SLC7A7, ITM2A, CD68, CD69, ICOS, FCGR1A, HLA-DRB5, LTF, IL2RG, SUCNR1, THBS1, ERV3, CD27, PLTP, ICAM1, STS, CR1, CR2, GPR18, KL, CMKLR1, CECR1, SLAMF7,

					LILRB1, TNFRSF9, CD38, SIRPG, P2RY10, TNFSF13B, CD274, PLA2G7
SP_PIR_KEYWORDS	cell membrane	23	2.42	0.009	GAPT, OR2L8, ITK, GBP5, GPR18, KL, CMKLR1, SKAP1, CD74, SLC7A7, RAB31, P2RY10, CD68, TNFSF13B, FCGR1A, ICOS, LILRB4, CD274, RAP1B, SUCNR1, OSTALPHA, SCAMP5, GBP1
Cluster 5	Enrichment Score: 2.34				
Category	Term	Count	%	PValue	Genes
GOTERM_BP_FAT	GO:0002684~positive regulation of immune system process	13	1.37	1.78E-07	ICAM1, CR1, CR2, SKAP1, CD74, CD38, SIRPG, SH2D1A, TNFSF13B, IL2RG, THBS1, CD27, SYK
GOTERM_BP_FAT	GO:0002696~positive regulation of leukocyte activation	8	0.84	1.31E-05	CD38, SIRPG, TNFSF13B, IL2RG, THBS1, CD74, CD27, SYK
GOTERM_BP_FAT	GO:0050867~positive regulation of cell activation	8	0.84	1.77E-05	CD38, SIRPG, TNFSF13B, IL2RG, THBS1, CD74, CD27, SYK
GOTERM_BP_FAT	GO:0002694~regulation of leukocyte activation	9	0.95	3.10E-05	CD38, SIRPG, TNFSF13B, CD274, IL2RG, THBS1, CD74, CD27, SYK
GOTERM_BP_FAT	GO:0050865~regulation of cell activation	9	0.95	4.52E-05	CD38, SIRPG, TNFSF13B, CD274, IL2RG, THBS1, CD74, CD27, SYK
GOTERM_BP_FAT	GO:0051251~positive regulation of lymphocyte activation	7	0.74	8.05E-05	CD38, SIRPG, TNFSF13B, IL2RG, CD74, CD27, SYK
**GOTERM_BP_FAT**	**GO:0050871~positive regulation of B cell activation**	**5**	**0.53**	**9.76E-05**	**CD38, TNFSF13B, IL2RG, CD27, SYK**
GOTERM_BP_FAT	GO:0051249~regulation of lymphocyte activation	8	0.84	1.11E-04	CD38, SIRPG, TNFSF13B, CD274, IL2RG, CD74, CD27, SYK
GOTERM_BP_FAT	GO:0050864~regulation of B cell activation	5	0.53	5.38E-04	CD38, TNFSF13B, IL2RG, CD27, SYK
GOTERM_BP_FAT	GO:0045579~positive regulation of B cell differentiation	3	0.32	0.001	IL2RG, CD27, SYK
GOTERM_BP_FAT	GO:0050863~regulation of T cell activation	6	0.63	0.002	SIRPG, TNFSF13B, CD274, IL2RG, CD74, SYK
GOTERM_BP_FAT	GO:0045621~positive regulation of lymphocyte differentiation	4	0.42	0.002	IL2RG, CD74, CD27, SYK
GOTERM_BP_FAT	GO:0050870~positive regulation of T cell activation	5	0.53	0.002	SIRPG, TNFSF13B, IL2RG, CD74, SYK

GOTERM_BP_FAT	GO:0051094~positive regulation of developmental process	8	0.84	0.005	XRCC2, MSR1, KL, IL2RG, THBS1, CD74, CD27, SYK
GOTERM_BP_FAT	GO:0045577~regulation of B cell differentiation	3	0.32	0.006	IL2RG, CD27, SYK
Cluster 6	Enrichment Score: 2.2				
Category	Term	Count	%	PValue	Genes
INTERPRO	IPR003597:Immunoglobulin C1-set	6	0.63	7.86E-05	SIRPG, HLA-DRB1, HLA-DRB5, IGK@, HLA-DMB, SIRPB1
SMART	SM00407:IGc1	6	0.63	1.37E-04	SIRPG, HLA-DRB1, HLA-DRB5, IGK@, HLA-DMB, SIRPB1
INTERPRO	IPR013783:Immunoglobulin-like fold	12	1.26	9.19E-04	LILRB1, ICAM1, SIRPG, HLA-DRB1, ICOS, FCGR1A, LILRB4, CD274, HLA-DRB5, IGK@, HLA-DMB, SIRPB1
INTERPRO	IPR007110:Immunoglobulin-like	11	1.16	0.002	LILRB1, SIRPG, HLA-DRB1, FCGR1A, LILRB4, CD274, HLA-DRB5, IGK@, SLAMF7, HLA-DMB, SIRPB1
INTERPRO	IPR003006:Immunoglobulin/major histocompatibility complex, conserved site	5	0.53	0.002	SIRPG, HLA-DRB1, HLA-DRB5, IGK@, HLA-DMB
INTERPRO	IPR003599:Immunoglobulin subtype	8	0.84	0.006	LILRB1, ICAM1, SIRPG, FCGR1A, LILRB4, CD274, SLAMF7, SIRPB1
SP_PIR_KEYWORDS	Immunoglobulin domain	9	0.95	0.007	LILRB1, ICAM1, SIRPG, ICOS, FCGR1A, LILRB4, CD274, SLAMF7, SIRPB1
Cluster 7	Enrichment Score: 2.18				
Category	Term	Count	%	PValue	Genes
KEGG_PATHWAY	hsa04640:Hematopoietic cell lineage	6	0.63	0.001	CD38, CR1, CR2, HLA-DRB1, FCGR1A, HLA-DRB5
Cluster 8	Enrichment Score: 2.14				
Category	Term	Count	%	PValue	Genes
INTERPRO	IPR003597:Immunoglobulin C1-set	6	0.63	7.86E-05	SIRPG, HLA-DRB1, HLA-DRB5, IGK@, HLA-DMB, SIRPB1
SMART	SM00407:IGc1	6	0.63	1.37E-04	SIRPG, HLA-DRB1, HLA-DRB5, IGK@, HLA-DMB, SIRPB1
GOTERM_BP_FAT	GO:0019882~antigen processing and presentation	6	0.63	3.67E-04	ICAM1, HLA-DRB1, HLA-DRB5, HLA-DMB, CD74, PSMB9
INTERPRO	IPR013783:Immunoglobulin-like fold	12	1.26	9.19E-04	LILRB1, ICAM1, SIRPG, HLA-DRB1, ICOS, FCGR1A, LILRB4, CD274, HLA-DRB5, IGK@, HLA-DMB, SIRPB1
KEGG_PATHWAY	hsa04672:Intestinal immune network for IgA production	5	0.53	0.001	TNFSF13B, HLA-DRB1, ICOS, HLA-DRB5, HLA-DMB
INTERPRO	IPR007110:Immu	11	1.16	0.002	LILRB1, SIRPG, HLA-DRB1, FCGR1A, LILRB4,

	noglobulin-like				CD274, HLA-DRB5, IGK@, SLAMF7, HLA-DMB, SIRPB1
GOTERM_BP_FAT	GO:0002504~antigen processing and presentation of peptide or polysaccharide antigen via MHC class II	4	0.42	0.002	HLA-DRB1, HLA-DRB5, HLA-DMB, CD74
UP_SEQ_FEATURE	region of interest:Beta-2	3	0.32	0.002	HLA-DRB1, HLA-DRB5, HLA-DMB
UP_SEQ_FEATURE	region of interest:Beta-1	3	0.32	0.002	HLA-DRB1, HLA-DRB5, HLA-DMB
INTERPRO	IPR003006:Immunoglobulin/major histocompatibility complex, conserved site	5	0.53	0.002	SIRPG, HLA-DRB1, HLA-DRB5, IGK@, HLA-DMB
INTERPRO	IPR000353:MHC class II, beta chain, N-terminal	3	0.32	0.005	HLA-DRB1, HLA-DRB5, HLA-DMB
KEGG_PATHWAY	hsa04612:Antigen processing and presentation	5	0.53	0.008	HLA-DRB1, TAP1, HLA-DRB5, HLA-DMB, CD74
KEGG_PATHWAY	hsa04514:Cell adhesion molecules (CAMs)	6	0.63	0.008	ICAM1, HLA-DRB1, ICOS, CD274, HLA-DRB5, HLA-DMB
INTERPRO	IPR014745:MHC class II, alpha/beta chain, N-terminal	3	0.32	0.009	HLA-DRB1, HLA-DRB5, HLA-DMB
Cluster 9	Enrichment Score: 1.11				
Category	Term	Count	%	PValue	Genes
GOTERM_BP_FAT	GO:0000279~M phase	9	0.95	0.003	KIF23, CCNB1, XRCC2, MKI67, PRC1, NCAPG, ANLN, KPNA2, ASPM
SP_PIR_KEYWORDS	cell division	7	0.74	0.005	KIF23, CCNB1, PRC1, NCAPG, CINP, ANLN, ASPM
SP_PIR_KEYWORDS	cell cycle	9	0.95	0.006	KIF23, CCNB1, MKI67, PRC1, NCAPG, CINP, E2F8, ANLN, ASPM
GOTERM_BP_FAT	GO:0022402~cell cycle process	11	1.16	0.009	KIF23, CCNB1, XRCC2, MKI67, PRC1, NCAPG, ANLN, THBS1, KPNA2, ASPM, PSMB9
GOTERM_BP_FAT	GO:0000279~M phase	9	0.95	0.003	KIF23, CCNB1, XRCC2, MKI67, PRC1, NCAPG, ANLN, KPNA2, ASPM

TableS7 Primer sequences for quantitative PCR

Primer	Sequence (5'→ 3')
**HBV**	
HBx+1550	CGTCTGTGCCTTCTCATC
HBx-1695	GGTCGGTCGTTGACATT
HBV+1811	ACCATGCAACTTTTTCACCTCTCTGCCTAATCATC
HBV-2016	CGATACAGAGCTGAGGCGGT
**Actin**	.
Beta-actin-F	GCTACGAGCTGCCTGACG
Beta-actin-R	GGCTGGAAGAGTGCCTCA
Universal probe #9	CATCACCA

TableS8 The customized gene set

Gene set	Genes
IFNα	ABCD1 ABTB2 ACHE ADAMTSL4 ADPRHL2 ANKFY1 APOBEC3B APOBEC3F ARHGAP27 ATXN7L1 BST2 C16orf70 C1orf38 CASP7 CCND3 CNP CSRNP1 CYP2J2 CYTH1 DDO EHD4 EPCAM FAM46A FAM46C FAM84B FTSJD2 GPR37 GTF2B H1F0 HERC5 HIST2H2AA3 HSH2D IFI6 IGFBP1 IL22RA1 IRF6 IRF7 ITPRIP KCTD5 KIAA0226 KIAA1217 KRT23 LCMT1 LGALS3BP MAB21L2 MAFK MASTL MICB MOBKL2C MSC MXD1 MYD88 N4BP1 NAPA OGFR PARP9 PHACTR4 PLEKHA4 PNPT1 POLA2 PRKD2 PRR15 RAB43 RAD9A RET RIPK1 S100A14 SDCBP2 SERPINB1 SERPINB2 SIDT1 SLC37A1 SMAD3 SP140L STK40 TACSTD2 THSD1 TICAM1 TINF2 TLR3 TMEM116 TMEM171 TMEM62 TMEM92 TNFRSF10A TRAF1 TRANK1 TRIM25 TRIM26 TRIM38 TRIM56 TUFT1 UBA7 UNC93B1 USF1 USP42 VTRNA1-3 YEATS2 ZBP1 ZFYVE26
IFNα∩γ	ΑΝΓΠΤΛ1 ΑΠΟΒΕΧ3Φ ΑΠΟΒΕΧ3Γ ΑΠΟΛ1 ΑΠΟΛ2 ΑΠΟΛ3 ΑΠΟΛ4 ΑΣΠΗΔ2 ΑΤΦ3 ΒΑ<br>ΤΦ2 ΒΧΛ2Λ14 ΒΤΝ3Α2 Χ19ορφ66 Χ4ορφ19 ΧΑΣΠ10 ΧΧΔΧ109Β ΧΧΛ8 ΧΧΡΛ1 ΧΔ274 ΧΔ<br>40 ΧΜΠΚ2 ΧΤΝΝΒΛ1 ΧΞ3ΧΛ1 ΧΞΧΛ10 ΧΞΧΛ11 ΧΞΧΛ9 ΧΨΠ1Α1 ΔΑΒ2ΙΠ ΔΔΞ58 ΔΔΞ60<br>ΔΔΞ60Λ ΔΕΝΝΔ5Α ΔΗΞ58 ΕΙΦ2ΑΚ2 ΕΝΔΟΔ1 ΕΠΣΤΙ1 ΕΡΑΠ2 ΕΤς6 ΕΤς7 ΦΑΜ122Χ ΦΛΤ<br>3ΛΓ ΦΣΤ ΓΑΛΜ ΓΒΠ3 ΓΒΠ4 ΓΒΠ5 ΓΜΠΡ ΓΤΠΒΠ1 ΗΧΠ5 ΗΕΓ1 ΗΕΡΧ6 ΗΡΑΣΛΣ2 ΙΔΟ1 Ι<br>ΦΙ16 ΙΦΙ27 ΙΦΙ35 ΙΦΙ44 ΙΦΙ44Λ ΙΦΙΗ1 ΙΦΙΤ1 ΙΦΙΤ2 ΙΦΙΤ3 ΙΦΙΤ5 ΙΦΙΤΜ1 ΙΛ15ΡΑ ΙΛ1ΡΝ ΙΡ<br>Φ1 ΙΡΦ2 ΙΣΓ15 ΙΣΓ20 ΙΤΠΚΧ ΚΙΑΑ0040 ΛΑΜΠ3 ΛΑΠ3 ΛΓΑΛΣ17Α ΛΓΑΛΣ9 ΛΓΑΛΣ9Β ΛΓ<br>ΑΛΣ9Χ ΛΙΝ52 ΛΨ6Ε ΜΓΧ39372 ΜΛΚΛ ΜΥΧ13 ΜΞ1 ΜΞ2 ΝΛΡΧ5 ΝΜΙ ΝΟΔ2 ΝΠΠΒ ΝΤ5<br>Χ3 ΝΥΑΚ2 ΝΥΒ1 ΟΑΣ1 ΟΑΣ2 ΟΑΣ3 ΟΑΣΛ ΠΑΡΠ14 ΠΔΧΔ1ΛΓ2 ΠΗΦ15 ΠΛΕΚΗΦ1 ΠΛΣ<br>ΧΡ1 ΠΜΛ ΠΡΙΧ285 ΠΡΟΧΡ ΠΣΜΒ9 ΡΝΔ1 ΡΝΦ114 ΡΝΦ122 ΡΝΦ19Β ΡΝΦ213 ΡΝΦ24 ΡΣΑ<br>Δ2 ΡΤΠ3 ΡΤΠ4 ΣΑΜΔ9 ΣΑΜΔ9Λ ΣΑΜΗΔ1 ΣΕΧΤΜ1 ΣΕΡΠΙΝΒ9 ΣΛΧ15Α3 ΣΛΧ25Α28 ΣΛΦ<br>Ν5 ΣΟΧΣ1 ΣΠ110 ΣΤΑΡΔ5 ΣΤΑΤ1 ΣΤΑΤ2 ΣΤΕΑΠ4 ΤΑΠ1 ΤΑΠ2 ΤΔΡΔ7 ΤΕΣΚ2 ΤΛΕ4 ΤΜΕ<br>Μ106Α ΤΜΕΜ110 ΤΜΕΜ140 ΤΜΠΡΣΣ3 ΤΝΦΑΙΠ3 ΤΝΦΣΦ10 ΤΝΦΣΦ13Β ΤΡΑΦΔ1 ΤΡΙΜ1<br>5 ΤΡΙΜ21 ΤΡΙΜ22 ΤΞΝΙΠ ΥΒΔ ΥΒΕ2Λ6 ΥΣΠ18 ΩΑΡΣ ΩΔΡ25 ΞΑΦ1 ΞΔΗ ΖΧ3ΗΑς1 ΖΝΦ38<br>5Β ΖΝΦΞ1
IFNγ	4-Sep AKAP12 ALPK1 ANO4 APOBEC3D APOL6 ARAP2 ARID5B ARNTL2 BAT5 BBX BCL6 BTN3A1 BTN3A3 C5orf56 CA13 CADPS2 CAND2 CARHSP1 CCDC68 CCL2 CCRN4L CD38 CD47 CD74 CD97 CDC25B CDCP1 CPA2 CTSO CTSS CYB561D2 CYP1B1 DAO DUOX2 DUOXA2 DUSP6 ELMO2 ELOVL7 FAM115C FAM117B FAM20A FBXO32 FBXO6 FRMD3 FRMD4B GABARAPL1 GBP1 GBP2 GBP7 GEM GLYAT GPR39 GPT2 GRAMD1C GSDMB HAPLN3 HLA-DMA HLA-DMB HLA-DOB HLA-DPA1 HLA-DPB1 HLA-DQB1 HLA-DRA HLA-DRB5 HLA-E ICAM1 IL18BP IRF8 JAK2 KLF4 KRT17 LGMN LHFPL2 LIMK2 LMO7 LPIN1 MDGA1 MOBKL2B MTHFR MUC1 NEURL3 NUDCD1 P2RX7 PAQR9 PARP8 PGLYRP4 PLA1A PLA2G2A PLSCR4 PMAIP1 PRRG1 PSMB10 PSMB8 PSME2 RAB31 RAG1AP1 RARRES1 RARRES3 RASSF5 RGS14 RGS7BP RIPK2 RPS6KA1 SERPINA7 SFMBT2 SIDT2 SLC2A12 SLC37A3 SOCS3 SP100 ST6GALNAC6 STARD8 STAT5A STS STXBP1 SYNE2 TEAD4 TMEM217 TRIM31 TRIM40 VNN2 VSNL1 XRN1 ZFP36 ZFP36L2 ZNF267
B cell	ADAM19 ADAM28 AF15Q14 AIM2 AKAP2 BACE2 BACH2 BCL10 BCL11A BCL2

associated genes	BCL7A BLR1 BRDG1 BSG BTK CAT56 CBFA2T1 CCR7 CD19 CD1C CD22 CD37 CD72 CD79A CD79B CD83 CEP1 CETP CKS2 CNR1 CNR2 COL6A2 CORO1A CR2 CREBBP CRKL CSF1 CYP19 DD5 DDX17 DHFR DLEU1 DMD DNAJB1 DTX1 E2F5 EBF EEF1G EGR1 ENTPD1 EPB41L2 EPHX1 FCER2 FLI1 FLJ20538 FOXO1A GL012 GSTT1 HDAC7B-PENDING HHEX HLA-DMB HLA-DNA HLA-DQA1 HNRPH1 HTR3A HUMHOXY1 ICSBP1 ID3 ID4 IFIT4 IGHG3 IGHM IGKC IGKV3D-15 IGL@ IGLL1 IGLL3 IL15RA IL7 INPP5A INPPL1 IRF4 IRTA1 IRTA2 ITGA2 ITPR1 KCNG1 KIAA0655 KIAA0868 KIAA1029 KLF1 KLF2 LARGE LOC51101 LOC51160 LOC55893 LOC56994 LY64 MAGEA5 MCM5 MEF2A MEF2C MGAT3 MOX2 MRPL36 MS4A2 MTX1 MUM2 MYO5C NCF1 NCOA3 NPIP NR1I3 NTT5 ODC1 PAX5 PCCA PCDH9 PKIG PMAIP1 PNOC POU2F2 POU4F1 PRDM2 PRKCE PSMB9 PTK2 PTPRN2 PTPRO RAB30 RalGPS1A RARA REL RGS16 RHO6 S100A5 SCYA19 1-Sep SERPINA1 SFRS2 SGCE SHMT2 Siat7d SLC2A1 SLC2A5 SLP65 SMARCA2 SP100 SP140 SPI1 SPIB SPRY1 STAG3 TAF2G TAP2 TCEB3 TCF3 TCF4 TCF6L1 TCF8 TCL1A TCL6 TIMELESS TLR10 TNFRSF17 TNFRSF5 TNFSF7 TPD52 TRAF4 TRIO TTK TULP1 UST VAV2 VI VIL2 VPREB3 WW45 ZFP161
T cell associated genes	ABLIM ADAM12 ADAM23 ADARB1 ADCY8 AKAP1 AKAP6 ALB ANK1 ANK3 ANKH AP3M2 APBB1 API5L1 AQP3 ATP1A1 B4GALT2 BCL11B BMP6 BNIP1 BNIP3 C4.4A C6 C8A CACNA1A CAMK4 CARS CASK CASP6 CBR3 CCNE1 CCR4 CD1A CD28 CD3D CD3E CD3G CD48 CD5 CD6 CD8A CDC14A CDH17 CDK5R1 CDR2 CDW52 CEBPD CHN1 CIN85 CNK COL6A1 CTLA4 CUL3 CYP2A7 CYP2E CYSLT1 D4S234E DGKA DNAJC6 DPP4 DSC1 DSCR1L2 DSS1 DXS6984E EGFL3 ENO2 EPHA1 EPHX2 ERBB3 ESR1 EWSR1 F5 FABP4 FAP FBLN5 FHIT FHL1 FKBP5 FLT3LG FMR2 FN1 FUT7 FVT1 FYB G6PD GAPL GAS41 GCHFR GFPT2 GOLGA2 GPA33 GPR18 GS3955 GYPC HFL1 HMIC HOXB3 HPIP IDUA IGF1R IKKE IL11RA IL14 IL6 IL6ST IL7R INPP4B INPP5D ITGA1 ITGA6 ITK JPO1 KCNA3 KI-1/57 KIAA0374 KIAA0855 KIF5C KITLG LAT LDHB LEF1 LMO7 LOC51029 LOC51669 LPL LTB LTK MAD2L1 MAF MAL MAPK3 MAPK8IP1 MATN2 MG61 MGAT4A MMP12 MRG15 MTCP1 MY047 MYB MYC NBL1 NDUFC1 NELL2 NET1 NR1D1 NR4A2 NT5 NTN4 P125 PAG PCOLN3 PDCD4 PDK1 PEX6 PIM1 PKIA PMS2 PMS2L2 POU6F1 PRAME PRDX2 PRG4 PRKCA PRKCL2 PTPN13 PXR2b RAB33A RASGRP1 RGS10 RORC RPS29 RPS4Y SCML1 SELL SELP SIAT8A SIT SLAM SLC11A3 SLC12A7 SNTA1 SNX9 SOD1 SPON1 SRI SRPK1 SUPT3H SVIL TACC3 T-cell TCF7 TFAP4 TIE TNF TNFRSF12 TNFRSF6 TNFRSF7 TNK1 TPM2 TPP2 TPR TRA@ TRADD TRB@ TRIM TYMSTR VIPR1 WNT7A ZNF140
NK cell associated	ABCB1 ACK1 ACLY ACTN4 ADAM8 ADAMTS1 ADRB2 AHCP AKR1C1 AKR1C2 AKR1C3 ALDH5A1 ANGPT2 APOA1 APS ARHGEF9 ATF1 ATP2B4 AXIN1 B3GAT1

genes	BATF BFSP1 BHLHB2 BMPR1A C5 CACNA2D2 CACNB2 CD97 CDKN2D CEBPG CLCN4 CLECSF2 CST7 CTSC CTSE CX3CR1 DAPK2 DLG5 DOC-1R ENPP4 ENPP5 EPB72 ERBB2 F2R FANCA FEZ1 FGR FIP2 GALNT3 GAS1 GCNT1 GFI1 GNG2 GOLPH2 GPR56 GTF3C1 GW128 GZMB Gzmc HEF1 HM74 HSPCA ID2 IFRG28 IGFBP7 IGSF4 IKBKB IL2RB IL8RB ITGAL Kifap3 KLRC1 KLRC2 KLRC3 KLRD1 LAK-4P LOC51020 LOC51226 LOC51738 LOC56939 MAD4 MATK MDS019 MNAT1 MYBL1 MYO6 MYOM2 MYPT1 NBS1 Ncam NCAM1 NPC1 NPDC1 PAFAH2 PAK6 PCAF PCTK2 PCYT2 PEX11A PGM5 PHEMX PI3 PIG7 PKM2 PM5 PON2 PPP2R2B PRAX-1 PRF1 PRSS12 PTCH PTGER4 PTK7 PTPN22 PTPN4 PTPN7 PTPRA PTPRM RAB27A RABIF RAG1 RAGE RALGDS RAP1GA1 RAP2A RASSF1 RGS3 RGS9 RUNX3 S100B SCYC1 SERPINB7 SLC14A1 SLC20A1 SLC4A4 SNTB2 SPAK SPUVE SRP14 SRP46 ST5 SUCLG2 SYNGR1 TBX21 TERT TFDP2 TGFBR3 TKTL1 TLR3 TMEM1 TNFSF6 TRAJ61 TRG@ TRIP7 UAP1 UBE2A VCL VDUP1 YES1 ZNF144 ZNF80
Chimpanzees_HBV	ADA AKR1B1 ANXA2 APOL3 ICB-1 B2M BTN3A3 C3AR1 CHST6 CECR1 CTSB CTSC CCL5 CD3D CD38 CD48 CD5L CD53 CD68 CD74 CD83 CLN2 CFL1 C1QB CXCL10 CXCL11 CXCL9 CXCR4 EMR1 EST FLJ11259 EST EST EST FcgRI FGL2 LGALS3 GM2A GZMA GZMK GBP1 GBP2 HLA-A HLA-B HLA-C HLA-DMA HLA-DMB HLA-DPA1 HLA-DPB1 HLA-DQB1 HLA-DRA HLA-DRB3 HLA-E IFI16 IFI27 IFI30 ISG20 IGHG3 IGLJ3 IGSF6 Z39IG FCER1G IL10RA IAN4L1 KPNA2 LAP3 LPXN LAPTM5 LYZ MCM6 PPT1 PIK3CD PTTG1 PLAC8 PRC1 PRG1 PSMB10 PSMB9 PSME1 PSME2 RAB20 RAB27A RAB31 RAC2 RGL RFX5 RARRES3 ARHGDIB RNASE6PL RRM2 S100A10 STK6 SLC7A7 SP110b STAT1 SOD2 TcR-B TcR-GC2 TcR-G TAPBP TYMS TOPK GPNMB TRIM22 WARS TYROBP UBD UBE2C UBE2L6 UCP2 OAS1
woodchuck_WHV	MMP7 PLA2G2A LOC96610 RRM2 IGLL3P CCNA2 PPBP CDKN1A CDK1 CCNB2 SLC47A1 EXO1 LCN2 TOP2A ORC1 KIF11 F13A1 CXCL9 LGALS3 SHCBP1 CENPE CDC20 PRR11 DLGAP5 POSTN BUB1B CENPF AURKB KIAA0101 PBK ARHGAP11A MMRN1 IGLL1 SFTPA1 SCIN ASPM CKAP2 MELK KIF20A UBE2C LTF ECT2 PAPOLA CDKN2C PLK1 CPM CDC6 NCAPG KIF2C MKI67 MYBL2 RGS1 THBS1 CDCA2 MIR650 FAP CXCR4 ANLN BUB1 F2RL2 PFKFB3 COL1A2 KIF23 CCNB1 HIST1H1D KIF15 CA1 TRIM59 SERPINB10 MPZL2 PRC1 ANXA1 TPX2 PLEK RGS7 TREM1 EGR2 KANK1 TIMP1 CCND1 SULF2 ICA1 DSCC1 BHLHA15 MYB MTHFD2 SPC25 TUBB1 MCM4 SLFN11 CFTR GPNMB FPR1 ANXA2 DTL HCN2 NCAPH ART4 ORC6 SRGN EPB41L3 CHAF1B KCNA3 LAIR1 IGJ RACGAP1 ALOX12 ITGA2B ITGB3 CHL1 SLFN13 NDC80 SLC2A3 SYT11 SLC15A2 SPECC1 BLM GPR65 S100A4 LUM CLEC5A EPCAM DIAPH3 RAD54L IL2RA STX11 ATP10A MSR1 EMILIN2 MARCO PIK3CG RAB27A VNN2 ZWILCH CDCA3 FANCD2 C6orf25 MYOF RNASE3 C5AR1 CHRNE GPR183 SMC4 NRGN CDC45 COL1A1 NAIP SYK WDHD1 VSIG4 KIFC1 IRF4 ST6GALNAC2 GCET2 BCL2A1 EZH2 CD48 PIK3R5 MCM5 CCNE2 PPAPDC1B CLP1 CLEC7A BIRC3 POLQ MST4 MME KNTC1 MCM3 SGOL2 RAD18 QPCT TCF19 STK39 FAT1 MPEG1 HPSE CD83 BASP1 EMBP1 CA11 MZB1 PLS1 INSL5 STIL CIT MGP CD4 MIS18BP1 CCR2 TRPS1 SLC38A1 CLDN7 RNASE6 TNFAIP3 EMB SDF2L1 SGOL1 RUNX1 MRVI1

	RGS2 RBL1 DNA2 ZNF367 GEM PTPN22 DEPDC1B C17orf87 VIM HK2 LAMP3 IL2RG CCR1 ALOX5 TSPYL2 CTSK FYB PLP2 OXCT1 BTK LAPTM5 ARHGAP25 CD84 FXYD2 MICAL1 CCR5 TAGLN CD200R1 LAMA2 IL7R CLEC4A CTSS CGREF1 PLXDC2 SAMSN1 NFATC4 NAV1 PLOD2 SEMA4F MMP14 LRMP RAD51 CXCR2 SIRPB1 MAFF RWDD2B ELOVL7 TIAM1 C4orf47 PTGDS CDK6 RTN4 SCN1B TAP1 LOC100507705 OASL SPAG5 GLIPR1 RIPK3 CRELD2 EMP3 SERPINB1 AGAP2 PTAFR BRCA1 LOC441666 NCOA7 EGR1 FKBP1B ALPK1 CD300LF S100A6 HLA-DPA1 CECR1 LOC100505790 DZIP1 TMEM156

Supplemental References

[1] Yeh SH, Tsai CY, Kao JH, Liu CJ, Kuo TJ, Lin MW, et al. Quantification and genotyping of hepatitis B virus in a single reaction by real-time PCR and melting curve analysis. J Hepatol 2004;41:659-666.

[2] Xiao C, Qin B, Chen L, Liu H, Zhu Y, Lu X. Preactivation of the interferon signalling in liver is correlated with nonresponse to interferon alpha therapy in patients chronically infected with hepatitis B virus. Journal of viral hepatitis 2012;19:e1-10.

[3] Jansen L, de Niet A, Makowska Z, Dill MT, van Dort KA, Terpstra V, et al. An intrahepatic transcriptional signature of enhanced immune activity predicts response to peginterferon in chronic hepatitis B. Liver Int 2014.

[4] Sarasin-Filipowicz M, Oakeley EJ, Duong FH, Christen V, Terracciano L, Filipowicz W, et al. Interferon signaling and treatment outcome in chronic hepatitis C. Proc Natl Acad Sci U S A 2008;105:7034-7039.

[5] Sarasin-Filipowicz M, Oakeley EJ, Duong FHT, Christen V, Terracciano L, Filipowicz W, et al. Interferon signaling and treatment outcome in chronic hepatitis C. Proceedings of the National Academy of Sciences 2008;105:7034-7039.

[6] Subramanian A, Tamayo P, Mootha VK, Mukherjee S, Ebert BL, Gillette MA, et al. Gene set enrichment analysis: a knowledge-based approach for interpreting genome-wide expression profiles. Proc Natl Acad Sci U S A 2005;102:15545-15550.

[7] Huang da W, Sherman BT, Lempicki RA. Systematic and integrative analysis of large gene lists using DAVID bioinformatics resources. Nature protocols 2009;4:44-57.

[8] Tian L, Greenberg SA, Kong SW, Altschuler J, Kohane IS, Park PJ. Discovering statistically significant pathways in expression profiling studies. Proc Natl Acad Sci U S A 2005;102:13544-13549.

[9] Waddell SJ, Popper SJ, Rubins KH, Griffiths MJ, Brown PO, Levin M, et al. Dissecting interferon-induced transcriptional programs in human peripheral blood cells. PLoS One 2010;5:e9753.

[10] Dill MT, Makowska Z, Duong FH, Merkofer F, Filipowicz M, Baumert TF, et al. Interferon-gamma-stimulated genes, but not USP18, are expressed in livers of patients with acute hepatitis C. Gastroenterology 2012;143:777-786 e771-776.
[11] Fletcher SP, Chin DJ, Ji Y, Iniguez AL, Taillon B, Swinney DC, et al. Transcriptomic analysis of the woodchuck model of chronic hepatitis B. Hepatology 2012;56:820-830.
[12] Wieland S, Thimme R, Purcell RH, Chisari FV. Genomic analysis of the host response to hepatitis B virus infection. Proc Natl Acad Sci U S A 2004;101:6669-6674.
